# Effectiveness of Physical Exercise Programs in Reducing Secondary Lymphedema Associated with Breast Cancer: An Overview of Systematic Reviews

**DOI:** 10.3390/jcm15135001

**Published:** 2026-06-26

**Authors:** Raúl Alberto Aguilera-Eguía, Carlos Zaror, Ruvistay Gutiérrez-Arias, Héctor Fuentes-Barria, Olga Patricia López-Soto, Cherie Flores-Fernández, Miguel Ángel Alarcón-Rivera, Ángel Roco-Videla, Bárbara Búrgos-Mansilla, Constanza Ulloa-López, Víctor Pérez-Galdavini, Luis Arriagada-Pérez, Mariana Melo-Lonconao, Xavier Bonfill, Pamela Seron

**Affiliations:** 1 Departamento de Salud Pública, Facultad de Medicina, Universidad Católica de la Santísima Concepción, Concepción 4090541, Chile; 2 Doctorado en Metodología de la Investigación Biomédica y Salud Pública, Universidad Autónoma de Barcelona, 08193 Barcelona, Spain; 3 Departamento de Odontopediatría y Ortodoncia, Facultad de Odontología, Universidad de La Frontera, Temuco 4811230, Chile; carlos.zaror@ufrontera.cl; 4 Center for Research in Epidemiology, Economics and Oral Public Health (CIEESPO), Faculty of Dentistry, Universidad de La Frontera, Temuco 4811230, Chile; xbonfill@santpau.cat; 5 Centro de Excelencia CIGES, Universidad de La Frontera, Temuco 4811230, Chile; 6 Departamento de Apoyo en Rehabilitación Cardiopulmonar Integral, Instituto Nacional del Tórax, Santiago 7500921, Chile; ruvistay.gutierrez@gmail.com; 7 Exercise and Rehabilitation Sciences Laboratory, School of Physical Therapy, Faculty of Rehabilitation Sciences, Universidad Andres Bello, Santiago 7591538, Chile; 8 INTRehab Research Group, Instituto Nacional del Tórax, Santiago 8580745, Chile; 9 Centro de Investigación en Medicina de Altura (CEIMA), Universidad Arturo Prat, Iquique 1110939, Chile; hefuentes_@unap.cl; 10 Departamento de Salud Oral, Facultad de Salud, Universidad Autónoma de Manizales, Pereira 660003, Colombia; sonrie@autonoma.edu.co; 11 Departamento Gestión de la Información, Universidad Tecnológica Metropolitana, Santiago 7550000, Chile; cflores@utem.cl; 12 Escuela de Ciencias del Deporte y Actividad Física, Facultad de Salud, Universidad Santo Tomás, Talca 3460000, Chile; mrivera3@santotomas.cl; 13 Facultad de Medicina, Universidad Católica del Maule, Talca 3480112, Chile; 14 Dirección de Desarrollo y Postgrados, Universidad Autónoma de Chile, Santiago 7500912, Chile; angel.roco@uautonoma.cl; 15 Departamento de Ciencias de la Rehabilitación, Facultad de Medicina, Universidad de La Frontera, Temuco 4811230, Chile; barbara.burgos@ufrontera.cl (B.B.-M.); constanza.ulloa@ufrontera.cl (C.U.-L.); 16 Departamento de Ciencias Clínicas y Preclínicas, Facultad de Medicina, Universidad Católica de la Santísima Concepción, Concepción 4090541, Chile; victorperez@ucsc.cl (V.P.-G.); larriagada@ucsc.cl (L.A.-P.); 17 Departamento Nacional de Salud Pública, Facultad de Medicina, Universidad San Sebastián, Sede Concepción, Concepción 4081339, Chile; mariana.melo@uss.cl; 18 Clinical Epidemiology and Public Health, Biomedical Research Institut Sant Pau, 08025 Barcelona, Spain

**Keywords:** breast neoplasms, lymphedema, breast cancer-related lymphedema, exercise therapy, physical therapy modalities

## Abstract

**Introduction**: Breast cancer-related lymphedema (BCRL) is a disabling complication, and the effectiveness of exercise as treatment remains uncertain. Objective: We aimed to evaluate the effectiveness and safety of exercise in women with BCRL. **Methods**: A comprehensive search was conducted in MEDLINE, Embase, Cochrane Library, PEDro, and LILACS from database inception to March 2025, collating systematic reviews (SRs) of randomized controlled trials (RCTs) evaluating exercise, alone or combined with physiotherapy, in women with BCRL. Risk of bias was assessed using ROBIS and RoB 2, certainty of evidence (CoE) using GRADE, and overlap using GROOVE. **Results**: Of 2023 records, 9 SRs including 170 primary studies were included; after overlap management, eligible RCT data were synthesized by comparison and outcome. Supervised weightlifting probably reduced the risk of a ≥5% long-term increase in lymphedema volume (RR: 0.13; 95% CI: 0.07–0.25; moderate CoE), but evidence is very uncertain regarding ≥5% volume reduction (RR: 0.85; 95% CI: 0.44–1.66; very low CoE). Aquatic exercise may improve shoulder flexion (MD: 8.73 degrees; 95% CI: 3.55–13.91; low CoE) and shoulder abduction (MD: 6.87 degrees; 95% CI: 2.50–11.24; low CoE) compared with Pilates. No adverse events were reported, although they were not systematically defined or reported. **Conclusions**: Supervised weightlifting probably prevents increases in lymphedema volume, and aquatic exercise may improve shoulder mobility; however, the evidence remains uncertain, and the absence of adverse events does not confirm safety. Registration: PROSPERO (CRD42022334433).

## 1. Introduction

Breast cancer is the most diagnosed cancer worldwide [1], while in high-income countries, mortality has declined due to advances in early detection and treatment [1,2], its incidence continues to rise with improved diagnostic methods, and although survival has increased, treatments are associated with adverse effects such as cardiotoxicity, neuropathy, fatigue, cognitive decline, and breast cancer-related lymphedema (BCRL) [3,4,5,6,7,8].

BCRL is one of the most underestimated and debilitating complications of breast cancer treatment [9]. Its incidence ranges from 3% to 65%, depending on treatment type and follow-up duration, and is mainly attributed to lymphatic flow interruption associated with mastectomy, axillary dissection, radiotherapy, taxane use, and obesity [10,11,12,13,14,15]. Clinically, BCRL manifests as swelling, heaviness, stiffness, restricted movement, pain, or discomfort; in severe cases, it can cause fibrosis and infections, significantly impairing quality of life (QoL) [16,17].

The treatment of BCRL is based on a multimodal approach where physiotherapy plays a fundamental role [18], including strategies such as complex decongestive therapy [19], manual lymphatic drainage [20,21], low-level laser therapy [22,23], pneumatic pumps [24], kinesiotaping [25,26], electrotherapeutic interventions such as transcutaneous electrical nerve stimulation and neuromuscular electrical stimulation, yoga [27], Pilates [27], and aquatic therapy [28]. Among these strategies, physical exercise programs have become relevant as part of physiotherapeutic BCRL treatment, with different approaches including aerobic exercise (AE) [29], resistance exercise (RE) [29], and combined programs [30]. Current clinical recommendations generally support the cautious and progressive use of supervised exercise as part of BCRL management, although the optimal modality, dose, intensity, and combination with other physiotherapeutic interventions remain uncertain. In this overview, “physical exercise programs” is used as an umbrella term to refer to structured exercise-based interventions evaluated in systematic reviews (SRs), including supervised weightlifting, resistance exercise, aerobic exercise, yoga, Pilates, aquatic exercise, and exercise combined with other physiotherapeutic modalities. These interventions were not assumed to be clinically or mechanistically interchangeable; therefore, findings were summarized and interpreted according to exercise modality, comparator, population, and outcome whenever data allowed. Numerous SRs have evaluated the effectiveness of physical exercise programs in the treatment of BCRL, but the results are heterogeneous and, on occasion, contradictory, which hinders a clear interpretation of the evidence [28,29,31,32,33,34,35,36,37,38,39,40,41,42,43,44,45,46,47].

From a biological perspective, exercise may influence lymphatic function through muscle-pump activity, increased blood flow, changes in interstitial fluid dynamics, improved upper-limb mobility, and enhanced lymph propulsion through intrinsic and extrinsic mechanisms. However, these mechanisms should be interpreted as physiologically plausible explanations rather than definitive evidence of clinical effectiveness.

Although previous reviews have examined exercise-based interventions for BCRL, the evidence remains difficult to interpret because SRs often overlap in their included primary studies, evaluate heterogeneous exercise modalities and comparators, and vary in methodological quality and certainty of evidence (CoE) assessment. Overlap among SRs is particularly relevant because repeated inclusion of the same primary studies can lead to double counting, overrepresentation of certain trials, and potentially misleading conclusions if not explicitly assessed and managed.

To our knowledge, no previous overview has focused specifically on SRs of RCTs evaluating physical exercise programs for established BCRL while explicitly assessing overlap among reviews and applying GRADE to the re-analyzed bodies of evidence.

An overview of reviews is appropriate for this purpose because it allows evidence from more than one SR of different interventions for the same condition or population to be summarized and critically appraised. Overviews enable comprehensive evidence synthesis and assessment of heterogeneity, bias, and methodological quality, thereby improving the CoE and supporting informed decision-making. Recently, Rafn et al. [48] published an overview evaluating various treatments for BCRL, including exercise, laser therapy, acupuncture, kinesiotaping, manual lymphatic drainage, and decongestive physiotherapy. Although their review provides valuable insights, it does not analyze SR overlap or the specific effects of physical exercise programs compared with other interventions. Moreover, the authors neither applied the GRADE approach to assess the CoE nor adjusted their conclusions according to the included studies’ quality.

This study addresses these limitations by including only SRs and meta-analyses of RCTs evaluating physical exercise programs, alone or combined with physiotherapeutic interventions, in women with established BCRL. The added value of this overview lies in its focused assessment of structured exercise programs for established BCRL, including exercise-based interventions delivered alone or in combination with physiotherapy. In addition, this overview provides an explicit assessment and management of overlap among SRs, re-analysis of non-duplicated RCT outcome data by comparison and outcome of interest, and the use of GRADE to assess the CoE of clinically relevant outcomes. The analysis focuses on effects on lymphedema volume, pain, and QoL, with secondary outcomes including adverse events, grip strength, range of motion, and upper-limb function.

## 2. Materials and Methods

### 2.1. Protocol and Registration

This overview followed the Cochrane Handbook for Systematic Reviews of Interventions [49] and the PRIOR statement checklist [50] (see Supplementary Material S1). The protocol was registered in PROSPERO (CRD42022334433) and published [51], and study selection was reported using the PRISMA flow diagram [52]. Designed as an overview of SRs, these reviews were the unit of search and inclusion. As pre-specified in the published protocol, overlap among SRs was assessed and managed to avoid double counting of primary-study outcome data. No independent search for primary RCTs outside the included SRs was conducted.

### 2.2. Design Eligibility Criteria

SRs, with or without meta-analysis [53], that followed an explicit methodology [54] were included. Reviews were required to define clear objectives and inclusion/exclusion criteria, perform searches in at least two databases, and assess the risk of bias using validated tools [55]. Two reviewers independently verified these elements, resolving discrepancies by consensus. SRs conducted using rapid review methodology were also included [56], considered eligible only if they met the same pre-specified methodological criteria used to define SRs in this overview. Therefore, classification as a rapid review was not, by itself, sufficient for inclusion.

When SRs included multiple study designs, only separately reported RCT data were considered for extraction and synthesis. Protocols and scoping or narrative reviews were excluded, and only studies in English, Spanish, or Portuguese were included.

### 2.3. Participant and Context Eligibility Criteria

SRs were eligible if they included women with established breast cancer-related lymphedema (BCRL). When SRs included mixed populations, such as women with established BCRL and women at risk of developing BCRL, only RCT data corresponding to women with established BCRL were extracted and re-analyzed, provided that these were reported separately or could be clearly identified. Data from studies including only women at risk of BCRL were not included in the outcome synthesis.

### 2.4. Intervention Eligibility Criteria

SRs were included that dealt with any type of physical exercise program, such as resistance exercise, aerobic exercise, yoga, or Pilates, either individually or in combination with other physiotherapeutic interventions, such as complex decongestive therapy, manual lymphatic drainage, or low-level laser therapy.

### 2.5. Comparison Eligibility Criteria

We included SRs in which the comparison group received usual care without structured exercise, did not receive active treatment, or were treated with some other physiotherapeutic intervention that was not a physical exercise program.

### 2.6. Outcome Eligibility Criteria

We considered the following primary outcomes:

- Volumetric changes in the arm, evaluated in comparison with the unaffected side and expressed as total lymphedema volume, volume reduction, or percent reduction. Measurements were carried out using validated methods, such as water displacement volumetry, circumference measurement, bioimpedance, dual X-ray absorptiometry, or perometry (see Supplementary Material S2).
- Quality of life, measured with generic or specific validated self-reported scales (e.g., EORTC-QLQ-C30).
- Pain intensity, evaluated using validated self-reported scales, such as the numeric rating scale (NRS) or visual analogue scale (VAS).

We considered the following secondary outcomes:

- Adverse events of exercise programs, such as increased lymphedema volume and pain.
- Grip strength, evaluated using dynamometry in patients undergoing physiotherapeutic interventions.
- Range of motion, measured using goniometry in patients who received physiotherapy.
- Upper-extremity function, evaluated with generic or specifically validated self-report scales, such as the DASH scale (Disabilities of the Arm, Shoulder, and Hand).

### 2.7. Information Sources

A comprehensive search for SRs of RCTs was conducted in MEDLINE/PubMed, Embase, Cochrane Library, PEDro, and LILACS, from the inception date of each database until March 2025. The search strategy combined natural language and controlled vocabulary terms, including MeSH, Emtree, and DeCS terms adapted to each database. These included concepts relevant to breast cancer-related lymphedema; exercise therapy; physical therapy modalities; resistance training; aquatic therapy; systematic reviews; and meta-analyses. References from relevant SRs and included studies were also reviewed to identify additional sources. The complete database-specific search strategies, including the controlled vocabulary and free-text terms used, are provided in Supplementary Materials S3–S7.

### 2.8. Selection Process

The results retrieved from the different databases were exported to Rayyan software (web-based software; Rayyan Systems Inc., Cambridge, MA, USA) [57] (https://www.rayyan.ai/) (accessed on 15 April 2025). After removing duplicates, two reviewers independently selected the studies based on titles, abstracts, and full texts, with discrepancies resolved by consensus or with a third reviewer. Excluded studies were documented, along with their corresponding reasons for exclusion.

### 2.9. Data Extraction

Data extraction was independently performed by two researchers using a Microsoft Excel^®^ spreadsheet, with a third author verifying the information and resolving discrepancies. Extracted data included the following:

- Publication details: Authors, year, research team, institutions, countries, databases, and type of synthesis (qualitative or quantitative).
- Participant characteristics: Sample size and key demographics.
- Intervention details: Exercise modality, type (multicomponent or single component), intensity, dosage, and duration.
- Comparator groups: Description of control or comparison conditions.
- Outcomes of interest: Primary and secondary outcomes.
- Authors’ conclusions: Main conclusions reported by the SR authors.

For the quantitative re-analysis, outcome data were extracted only from eligible RCTs contained within the included SRs. These RCT data were extracted only when they addressed the overview research question, involved women with established BCRL, evaluated eligible physical exercise programs, and contributed data to at least one pre-specified outcome.

### 2.10. Derivation of the Analytic RCT Set

The primary-study records reported across the included SRs were first mapped in an overlap matrix, with duplicate records then removed to identify the unique primary-study set. Each unique record was assessed against the overview eligibility criteria, including study design, population, intervention, comparator, and outcome relevance. This approach was applied because some SRs had a broader scope than the overview question; therefore, only the subset of primary studies meeting the overview eligibility criteria was retained in the analytic set.

These retained RCTs were those that addressed the overview research question, included women with established BCRL, evaluated eligible physical exercise programs, and contributed data to at least one pre-specified outcome. Studies that did not meet these criteria were excluded, and the reasons for exclusion are provided in Supplementary Material S9. The final analytic RCT set was then organized by comparison, exercise modality, follow-up time, and outcome before quantitative or narrative synthesis.

### 2.11. SR Risk of Bias Assessment

Two researchers independently assessed the risk of bias using the ROBIS tool, following the Cochrane Handbook for Systematic Reviews of Interventions guidelines [58]. Discrepancies were resolved by consensus or with the involvement of a third reviewer.

The ROBIS tool was applied in three phases [58]:

1. Assessment of relevance.
2. Identification of concerns in the review process.
3. Judgement on the risk of bias.

Signalling questions were marked with either “yes” or “no information,” and overall bias was rated as “high,” “low,” or “unclear” [58].

### 2.12. Risk of Bias in Included Primary Studies

Since none of the included SRs assessed the primary-study risk of bias using the RoB 2 tool [59], a full RoB 2 assessment was conducted for the RCTs retained in the analytic set. This covered bias arising from the randomization process, due to deviations from intended interventions, due to missing outcome data, in outcome measurement, and in selection of the reported result. Each domain was classified as “low risk,” “some concerns,” or “high risk of bias” [59].

### 2.13. Certainty of Evidence in Included SRs

Only one of the included SRs reported the CoE using the GRADE approach. However, that evaluation was based on a risk of bias assessment using a different tool from RoB 2 (Effective Public Health Practice Project Quality Assessment Tool), making it incompatible with this overview’s methodological approach. Furthermore, the studies included in that review did not completely overlap with the RCTs retained in our analytic set.

For these reasons, we decided to carry out a complete and independent evaluation of the CoE for each relevant clinical outcome using the GRADE approach, which includes domains for study design, risk of bias (evaluated with RoB 2.0), inconsistency, indirect evidence, imprecision, publication bias, and other considerations. We generated tables containing our findings with GRADEpro (www.gradepro.org) (accessed on 6 August 2025), classifying CoE into four levels: high, moderate, low, or very low [60]. A partially contextualized approach was applied, using the null effect as the threshold to determine whether the interventions provided a meaningful benefit compared with the comparator [61,62].

Conclusions were formulated following the standardized language proposed by the GRADE Working Group, combining effect size with certainty level [61,62]:

- High certainty: The intervention has an effect.
- Moderate certainty: The intervention probably has an effect.
- Low certainty: The intervention may have an effect.
- Very low certainty: The evidence about the effect is very uncertain.

This approach facilitates clear communication of the findings, in line with current GRADE standards for SRs [62].

### 2.14. Overall Certainty Assessment of the Evidence in the Overview

For the assessment of the overall CoE, the GRADE approach [60] was applied, analyzing the effects of physical exercise on the outcomes of interest.

### 2.15. Managing Overlapping SRs

Primary-study overlap was identified within the included SRs; therefore, to avoid double counting, each study was considered only once [63]. Overlap was assessed using a visual matrix and the Corrected Covered Area (CCA), categorized as low (0–5%), moderate (6–10%), high (11–15%), or very high (>15%), and graphically represented with the GROOVE tool [64].

### 2.16. Data Synthesis

The results were reported according to the recommendations of the Cochrane Handbook for Systematic Reviews of Interventions [65]. As this study was an overview of SRs, these reviews were the unit of search and inclusion, and no independent search for primary RCTs outside the included SRs was conducted.

As pre-specified in the published protocol, when overlapping SRs were identified, the outcome data from the repeated RCTs were re-extracted and re-analyzed only once, avoiding double counting and providing more specific and consistent estimates for each comparison and outcome of interest. Primary studies were considered only insofar as they were contained within the included SRs and contributed data to the pre-specified outcomes. We also verified whether the RCTs contained in the included SRs addressed the overview research question, avoiding the inclusion of indirect evidence.

When appropriate, new meta-analyses were conducted for each comparison and outcome of interest. Subgroup analyses were planned according to the exercise modality or program, with the following effect measures used:

- Relative risk (RR) with a 95% confidence interval (CI) for dichotomous data.
- Mean difference (MD) with 95% CI for continuous outcomes measured on the same scale.
- Standardized mean difference (SMD) when outcomes were measured on different scales.

Heterogeneity was assessed using the I^2^ statistic, interpreted as considerable when above 75%, moderate between 40% and 75%, and low when below 40% [66].

Reporting bias was assessed if the meta-analysis included at least 10 RCTs, applying the Begg test to analyze the funnel plot [66]. In the case of asymmetry, other causes were examined, such as publication bias, selective result reporting, low methodological quality of small studies, and heterogeneity.

Sensitivity analysis was performed when the number of studies allowed, restricting it to those with low risk of bias, in order to evaluate the robustness and trustworthiness of the obtained results.

### 2.17. Use of Generative Artificial Intelligence

During the preparation of this manuscript, the authors used ChatGPT (OpenAI, GPT-5.4 Thinking) exclusively to support language editing, improve the clarity of the text, and optimize grammar and wording. The tool was not used to perform any methodological or analytical procedures, such as study selection, data extraction, data analysis, risk of bias assessment, result interpretation, or scientific conclusion formulation. After using this tool, the authors critically reviewed, edited, and approved the final content, taking full responsibility for the integrity and accuracy of the manuscript.

## 3. Results

### 3.1. Study Selection

**Figure 1:**
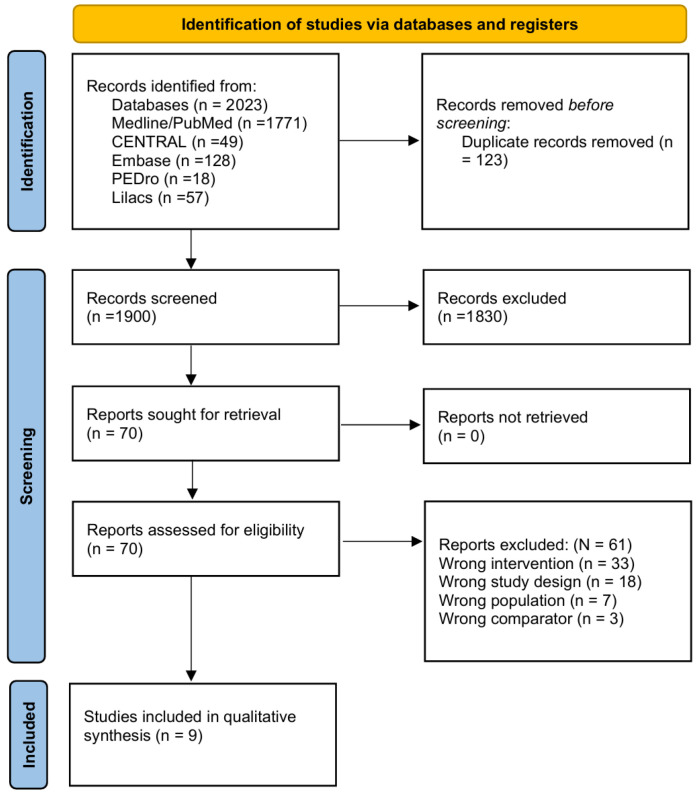
PRISMA 2020 flow chart of the study selection process.

The database search identified 2023 SRs or meta-analyses, with 1900 remaining after removing 123 duplicates. A further 1830 SRs were excluded by title and abstract screening, leaving 70 full texts to be assessed for eligibility. Finally, nine SRs were determined to meet the inclusion criteria [44,47,67,68,69,70,71,72,73]. The selection process is presented in the PRISMA flow diagram (Figure 1), while Supplementary Materials S8 and S9 provide details of the excluded reviews [19,20,21,23,26,29,33,34,35,36,48,74,75,76,77,78,79,80,81,82,83,84,85,86,87,88,89,90,91,92,93,94,95,96,97,98,99,100,101,102,103,104,105,106,107,108,109,110,111,112,113,114,115,116,117,118,119,120,121,122,123] and RCTs [124,125,126,127,128,129,130,131,132,133,134,135,136,137,138,139,140,141,142,143,144,145,146,147,148,149,150,151,152,153,154,155,156,157,158,159,160,161,162,163,164,165,166,167,168,169,170,171,172,173,174,175,176,177,178,179,180,181,182,183,184,185,186,187,188,189,190,191,192,193,194,195,196,197,198,199,200,201,202,203,204,205,206,207,208,209,210], respectively, and their reasons for exclusion. The primary RCTs included within the eligible SRs are cited in the main manuscript [211,212,213,214,215,216,217,218,219,220,221,222,223,224].

Across the nine included SRs, 170 primary-study records were initially identified and mapped in the overlap matrix, allowing duplicates to be removed, resulting in 105 unique primary-study records. These unique records were then assessed against the overview eligibility criteria, including study design, population, intervention, comparator, and outcome relevance, with only RCTs that addressed the overview research question, included women with established BCRL, evaluated eligible physical exercise programs, and contributed data to at least one pre-specified outcome retained for quantitative or narrative outcome synthesis. Studies that did not meet these criteria were excluded, with their reasons for exclusion reported in Supplementary Material S9. The final analytic RCT set was organized by comparison, exercise modality, follow-up time, and outcome before synthesis.

### 3.2. Characteristics of the Included SRs

Of the nine SRs included in the analysis, two were conducted in Australia (*n* = 2), while the remaining studies were distributed across Finland, Ireland, Spain, Switzerland, the United Kingdom, Thailand, and Iran. The earliest included SR was published in 2009 by Kärki [67], and among the eligible RCTs retained in the analytic set, the earliest primary study was that by McKenzie, published in 2003 [218]. The SRs analyzed between 10 and 36 studies each, and only two SRs reported the total participants included, with values ranging from 606 to 1091 participants. In terms of research design, the reviews evaluated both RCTs and non-RCTs, and no rapid reviews were included. The SRs primarily included patients with BCRL or at risk of developing it, as well as, in some cases, patients with upper-extremity lymphedema due to other types of cancer.

The physical exercise programs evaluated included therapeutic exercises delivered either as standalone interventions or in combination with other physiotherapeutic modalities, comprising compression bandaging, manual lymphatic drainage, pneumatic compression, low-level laser therapy, and electrical stimulation, as well as various exercise modalities such as yoga, resistance exercise, combined resistance and aerobic exercise, aquatic exercise, and weight training. Control groups were most frequently subject to non-exercise interventions such as usual care, self-care, or standard care, with or without non-structured activities (e.g., light exercise, gentle stretching). Other comparators reported in the included SRs were placebo or sham therapy, physiotherapeutic interventions such as compression bandaging or manual lymphatic drainage, unclassified physiotherapeutic interventions (e.g., land-based or aquatic physical activity not meeting the definition of a structured program), intervention control groups, sham exercise, and “other types of exercise” not matching the definition of the evaluated intervention. In some cases, the comparator consisted of a combination of treatments including exercise alongside other physiotherapeutic modalities. Although the eligibility criteria allowed comparators involving alternative non-exercise physiotherapeutic interventions, most of the included studies compared physical exercise programs (alone or in combination with other interventions) against no exercise, usual care, or non-structured activities—direct comparisons with alternative physiotherapy modalities as standalone interventions were infrequent.

The tools used to assess risk of bias varied between studies, with ROB 1 and PEDro being the most frequently applied (*n* = 3), followed by the Effective Public Health Practice Project Quality Assessment Tool (*n* = 2), while Jadad and the Guide to Community Preventive Services were used in one SR each. Table 1 and Supplementary Material S10 summarize these assessments’ main characteristics and key conclusions.

### 3.3. Evaluation of Included SRs’ Methodological Quality

The risk of bias of the nine SRs was analyzed across three phases: relevance, data management, and synthesis of findings. In Phase 1, all the SRs were considered relevant, with no problems identified in this domain. In Phase 2, most of the SRs presented a high risk of bias. Concerning eligibility criteria, six SRs were high risk [44,67,68,69,71,73], two were low risk [47,70], and one was uncertain [72]. Regarding the identification and selection of studies, nine SRs had high risk of bias, indicating inadequate search strategies, while in data collection and quality evaluation, seven SRs presented high risk, and one was uncertain, reflecting issues in primary-study extraction and assessment. In Phase 3, eight SRs presented high risk in the synthesis of the findings and only one had low risk. In the global bias evaluation, all the SRs (*n* = 9) were classified as high risk, indicating significant methodological limitations that may affect their results. Table 2 summarizes the risk of bias in the SRs across domains.

### 3.4. Degree of Overlap Between SRs

An overall overlap of 17.9% was identified, decreasing to 7.7% after applying adjustments to eliminate duplicate primary studies, indicating a moderate level of redundancy among SRs. Of the 170 primary studies initially extracted, 105 were retained after removing duplicates, with 36 studies included in more than one SR, reflecting that certain primary studies were repeatedly included across multiple SRs. Specifically, 20 studies were included in two SRs, 7 were found in three SRs, 6 appeared in four SRs, and 2 were identified in six SRs. Additionally, the overlap classification showed that 16, 7, 6, and 7 studies exhibited slight (<5%), moderate (≥5–<10%), high (10–<15%), and very high overlap (≥15%), respectively.

**Figure 2:**
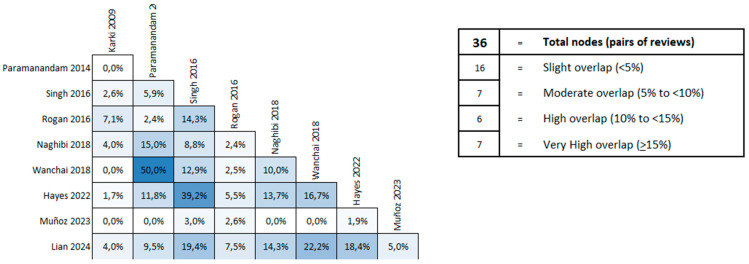
Graphical Representation of Overlap for Overviews (GROOVE) [64].

Supplementary Material S11 details, and Figure 2 illustrates, using GROOVE, the overlap among SRs and shared primary studies.

### 3.5. Certainty of Evidence Reported in Included SRs

The CoE evaluation across the nine SRs revealed a high level of methodological variability. Only three SRs applied specific CoE tools [67,71,72]—Karki (2009) utilized the Van Tulder scale [67], Singh (2016) applied the National Health and Medical Research Council criteria [71], and Hayes (2022) employed the GRADE approach [72]—while, in contrast, six SRs [44,47,68,69,70,73] did not evaluate CoE, compromising the interpretation and applicability of their findings.

Karki (2009) found limited evidence for most of the physiotherapeutic interventions evaluated [67], while Singh (2016) [71] found moderate to low CoE for the effects of exercise on lymphedema; although no adverse events were reported, the benefits were inconclusive across studies, and the certainty was downgraded due to imprecision. Hayes (2022) [72] reported moderate CoE for the prevention of lymphedema and very low to low CoE for a reduction in its volume in the treatment setting. The comparison between exercise and usual care showed the most consistent effects; however, in the treatment analysis, the CoE was mainly downgraded due to risk of bias and study design limitations. Table 1 provides a detailed description of the tools used in each SR.

### 3.6. Primary-Study Risk of Bias

A total of 14 eligible RCTs retained in the analytic set were assessed using the RoB 2 tool, with none of the RCTs judged to be at low overall risk of bias; eight were judged as having some concerns, and six were judged to be at high risk of bias. The studies judged as having some concerns were Jeffs (2013) [211], Schmitz (2009) [212], Do (2015) [215], Cormie (2013) [216], Kim (2010) [217], McKenzie (2003) [218], Odynets (2019) [222], and Şener (2017) [223], and those at a high risk of bias were Loudon (2014) [213], Pasyar (2019) [214], Tidhar (2010) [219], Speck (2010) [220], Letellier (2014) [221], and Johansson (2013) [224].

The included RCTs presented limitations across several RoB 2 domains. In Domain 1 (D1—bias arising from the randomization process), concerns were mainly related to deficiencies in the randomization process and allocation concealment, while in Domain 2 (D2—bias due to deviations from intended interventions) they were related to deviations from the intended interventions without adequate control strategies and limited use of intention-to-treat analysis. In Domain 3 (D3—bias due to missing outcome data), several studies did not adequately justify missing data or implement appropriate methods to address it, and in Domain 4 (D4—bias in measurement of the outcome), the absence of blinding and limited information regarding outcome measurement procedures represented additional concerns. In contrast, all studies were judged to be at low risk of bias in Domain 5 (D5—bias in selection of the reported result), indicating that the reported outcomes were consistent with those pre-specified or expected based on study protocols or registrations. Supplementary Material S12 presents a graphical summary of the RoB 2 assessment across domains for the primary studies.

### 3.7. Intervention Effects

#### 3.7.1. Primary Outcomes

A detailed summary of all effect estimates, confidence intervals, and CoEs for each outcome is provided in Supplementary Material S13. Overall, most outcome estimates were informed by one or a small number of RCTs with limited sample sizes; therefore, the findings are presented descriptively and should be interpreted with caution, particularly when comparing exercise modalities.

#### 3.7.2. Volumetric Changes in the Arm

- Lymphedema volume (LV) (<6 months): One SR [67], which included one RCT with 23 participants [211], was included in the meta-analysis, demonstrating uncertainty about whether a home-based physical exercise program combined with standard self-care may reduce LV compared with standard self-care alone. The CoE was very low.
- Lymphedema volume (LV) (>6 months): Six SRs [47,68,70,71,72,73] identified the same RCT with 139 participants [212]. After overlap management, this RCT contributed once to the meta-analysis and showed that a supervised weightlifting program probably reduces the risk of a ≥5% increase in LV compared with standard care (RR: 0.13; 95% CI: 0.07 to 0.25; moderate CoE). On the other hand, for a ≥5% reduction in LV, the evidence is very uncertain about the effect of the program (RR: 0.85; 95% CI: 0.44 to 1.66; very low CoE).
- Volume reduction (VR) (<6 months): One SR [68], which included one RCT with 23 participants [211], was included in the meta-analysis, and showed that there is uncertainty about whether a home exercise program in addition to standard self-care may reduce VR compared with standard care. The CoE was very low.
- Percent reduction (<6 months): One SR [68], which included one RCT with 23 participants [211], was included in the meta-analysis. The evidence is very uncertain regarding the effect of a home exercise program in addition to standard care on percentage reduction in lymphedema volume compared with standard care alone. The CoE was very low.

#### 3.7.3. Quality of Life (QoL)

- Global quality of life (<6 months): Five SRs [44,47,68,71,72] provided evidence, identifying four RCTs [211,213,214,215] with a total of 113 participants. The evidence is deeply uncertain about the effect of the interventions due to a very low CoE.
- Physical functioning (<6 months): Nine SRs [44,47,67,68,69,70,71,72,73] included six RCTs [214,215,216,217,218,219] with a total of 297 participants. The CoE was very low, leaving the effects of the interventions very uncertain.
- Role functioning (<6 months): Five SRs [44,47,68,71,72] contributed three RCTs [213,214,215] with a total of 90 participants. The certainty of the evidence was extremely low, and the effects remain highly uncertain.
- Emotional functioning (<6 months): Four SRs [47,68,69,72] identified three RCTs [214,215,219] including 119 participants in total. The CoE was very low, limiting confidence in the observed effects.
- Social functioning (<6 months): Seven SRs [44,47,68,69,70,72,73] contributed four RCTs [214,215,217,219] with 159 participants, some included in more than one SR. The CoE was very low, and the evidence on the effect of the interventions is very uncertain.
- Mental health (<6 months): Six SRs [44,68,70,71,72,73] identified two RCTs [216,217] with a total of 164 participants. The CoE was very low, generating substantial uncertainty regarding the effects.
- Mental health (>6 months): One SR [70] included a single RCT [220] with 112 participants that compared weight training to no intervention. The CoE was very low, making the evidence on the effect of the intervention very uncertain.
- Pain (<6 months): Four SRs [44,68,71,72] provided evidence, identifying two RCTs [213,221] with a total of 37 participants. The evidence is highly uncertain about the effect of the interventions due to very low CoE.

#### 3.7.4. Secondary Outcomes

A detailed summary of all effect estimates, confidence intervals, and CoEs for each outcome is provided in Supplementary Material S14.

- Adverse events (AEs): None of the included studies reported AEs. However, because they were not consistently defined or systematically reported across the included RCTs, the absence of reported AEs should not be interpreted as definitive evidence of safety.
- Grip strength (<6 months): Five SRs [44,68,71,72,73] contributed evidence toward this outcome. Across these reviews, two unique RCTs [216,221] were identified, with a total of 142 participants. The evidence is highly uncertain about the effect of the interventions due to a very low CoE.

Range of motion (ROM):

- Wrist and elbow ROM (<6 months): Four SRs [68,71,72,73] contributed evidence concerning this outcome, all identifying the same RCT [216] with 124 participants, which evaluated the effect of resistance exercise on wrist flexion and extension, as well as elbow flexion and extension. The evidence is notably uncertain about the effect of the intervention for all outcomes due to very low CoE.
- Shoulder flexion ROM (<6 months): Across five SRs [68,69,71,72,73] contributing evidence on this outcome, four RCTs were identified, with a total of 194 participants. One study [222] suggested that aquatic exercise may be more beneficial than Pilates (low CoE). For the other studies, the evidence is highly uncertain about the effect of the interventions due to a very low CoE [216,223,224].
- Shoulder extension ROM (<6 months): Four SRs [68,71,72,73] presented evidence on this factor, identifying two RCTs [216,222] with a total of 192 participants. One study [222] suggested that aquatic exercise may be more beneficial than Pilates (low CoE), while the other study [216] presented remarkably uncertain evidence regarding its intervention’s effect due to a very low CoE.
- Shoulder abduction ROM (<6 months): Five SRs [68,69,71,72,73] contributed evidence for this outcome across four RCTs with 277 participants in total. One study [222] suggested that aquatic exercise may be more beneficial than Pilates (low CoE), while in the other studies, the evidence is very uncertain about the effects of the interventions due to a very low CoE [216,223,224].
- Shoulder internal rotation ROM (<6 months): One SR [72] provided evidence on this factor, identifying a single RCT with 68 participants that compared aquatic exercise with Pilates (MD: 1.93; 95% CI: −1.05 to 4.91). The evidence is highly uncertain about the effect of the intervention due to very low CoE [222].
- Shoulder external rotation ROM (<6 months): Four SRs [68,69,71,72] addressed this outcome, identifying three RCTs [222,223,224] with a total of 153 participants. The evidence is very uncertain about the effect of the interventions due to a very low CoE.
- Upper-limb function (<6 months): Three SRs [44,47,72] provided evidence on upper-limb function, presenting three RCTs [215,221,223] with a total of 122 participants. The evidence is very uncertain about the effect of the interventions due to the very low CoE.

### 3.8. Results of Additional Analyses

Sensitivity, subgroup, and formal publication bias analyses were not conducted as none of the meta-analyses included the required minimum number of studies. In particular, publication bias or small-study effects could not be formally assessed using funnel-plot inspection or statistical tests for asymmetry because fewer than 10 RCTs contributed to each meta-analysis.

This limited number of studies included in each comparison and outcome also made a formal heterogeneity investigation impossible. Nevertheless, the main potential sources of clinical and methodological heterogeneity were identified across the included evidence, including differences in exercise modality, intervention dose and supervision, comparator type, follow-up time, BCRL measurement methods, outcome definitions, and risk of bias across RCTs. These heterogeneity sources were considered when deciding whether quantitative synthesis was appropriate and when interpreting the findings.

## 4. Discussion

### 4.1. Summary of Key Findings

This overview identified modality-specific signals of potential benefit from exercise-based interventions in women with established BCRL. Supervised weightlifting probably reduces the risk of long-term lymphedema volume increase (>6 months) (moderate CoE), whereas aquatic exercise may improve selected short-term shoulder range of motion outcomes (<6 months) compared with Pilates (low CoE). However, these findings should not be interpreted as evidence of a common effect across all physical exercise programs.

For most other modalities and outcomes, including resistance exercise, yoga, Pilates, multicomponent programs, QoL, pain, grip strength, upper-limb function, and several other range of motion measures, the evidence remains uncertain or very uncertain. This uncertainty is mainly explained by the small number of contributing RCTs, limited sample sizes, methodological limitations, and low or very low CoEs.

The lack of long-term follow-up in the included studies also limits the ability to draw definitive conclusions about sustained effects. Therefore, conclusions should be interpreted according to exercise modality, comparator, outcome, population, and follow-up time, rather than as broad comparative conclusions about physical exercise programs as a homogeneous intervention class. Although supervised weightlifting and aquatic exercise showed the clearest signs of potential benefit, these interventions should be considered with caution as part of individualized rehabilitation strategies. Given the high risk of bias in the included systematic reviews and the heterogeneity across studies, considerable uncertainty remains.

### 4.2. Clinical Implications

The clinical implications of this overview should be interpreted by exercise modality rather than for physical exercise programs as a single homogeneous intervention category. Even in supervised weightlifting and aquatic exercise, which tended most obviously toward potential benefit, these effects were outcome-specific and supported by limited evidence. Supervised weightlifting probably reduces the risk of long-term lymphedema volume increase, whereas aquatic exercise may improve selected short-term shoulder range of motion outcomes. Evidence for other exercise modalities, including resistance exercise, yoga, Pilates, and multicomponent programs, remains uncertain or very uncertain for most outcomes.

Therefore, exercise-based interventions should not be considered interchangeable therapeutic options. Their use should be individualized according to the specific exercise modality, clinical objective, patient characteristics, functional status, comorbidities, preferences, and availability of supervision. Given the limited CoE, these interventions should be considered with caution, preferably within supervised clinical or research settings, with realistic expectations and close monitoring of relevant outcomes.

While no adverse events were reported in the included studies, the absence of reported harms should not be interpreted as definitive evidence of safety. Adverse events in exercise trials may be inconsistently defined, incompletely reported, or treated as secondary observations. Therefore, clinical implementation should include active monitoring of potential adverse events, including increases in lymphedema volume, pain, and functional deterioration.

In individual studies, physical exercise programs have been associated with possible improvements in lymphedema reduction, muscle strength, function, mobility, QoL, and pain relief [29,31,32,225]. However, these effects were not consistently confirmed in our synthesis, and the CoE was mostly low or very low. Exercise may improve lymphatic function by increasing blood flow, cardiac output, and arterial pressure, thereby facilitating capillary filtration and fluid and protein absorption [38,226,227]. Additionally, lymph propulsion through lymphatic vessels may be aided by intrinsic and extrinsic mechanisms, such as muscle contractions, breathing, and arterial pulsation from nearby blood vessels [226,227]. Although these mechanisms provide physiological plausibility, they should be interpreted as hypothesis-generating rather than proof of clinical effectiveness.

Given these uncertainties, clinical application should proceed with caution, considering the lack of robust evidence for many outcomes and the heterogeneity in lymphedema assessment methods, which limit both the synthesis of results and their practical application. Standardizing clinical evaluation criteria is essential to enable result comparisons across studies and to ensure that therapeutic decisions are based on solid and reproducible evidence.

### 4.3. Implications for Future Research

This overview highlights the need for more rigorous and up-to-date SRs on the effects of exercise-based interventions in women with BCRL. Although this synthesis provides relevant information for health professionals, researchers, and patients, it also reveals important methodological gaps that should be addressed in future research.

All included SRs presented high risk of bias according to the ROBIS tool, mainly due to limitations in study eligibility criteria, search and selection processes, data collection, study appraisal, and synthesis of findings. This underlines the need for future SRs to be designed and reported according to recognized methodological standards, such as the Cochrane Handbook for Systematic Reviews of Interventions, the PRISMA guidelines, and the PRIOR statement for overviews [228,229,230,231]. Future reviews should also use appropriate tools, such as RoB 2, to assess the risk of bias of included RCTs, and should formally assess the certainty of evidence using the GRADE approach.

The predominance of low or very low CoE across most outcomes reflects important limitations in the primary RCTs, including small sample sizes, insufficient blinding, inconsistent outcome definitions, and heterogeneity in intervention and comparator characteristics. In light of this, future RCTs should address several key design deficits. First, diagnostic criteria and measurement methods for BCRL should be standardized, including clear definitions of lymphedema volume change, clinically meaningful thresholds, and consistent use of validated measurement methods such as bilateral limb volume assessment, perometry, bioimpedance spectroscopy, and standardized circumferential measurements. Second, adverse events should be prospectively defined, systematically monitored, and consistently reported, including worsening of lymphedema volume, pain, functional deterioration, and other exercise-related harms.

Third, future studies should clearly separate prevention and treatment populations. Trials evaluating women at risk of developing BCRL should not be analyzed together with trials enrolling women established as having the complication, because these represent different clinical questions. Fourth, control groups should be more clinically comparable and clearly defined, with comparisons such as exercise versus usual care, exercise versus no exercise, aquatic exercise versus Pilates, or exercise combined with physiotherapy versus physiotherapy alone analyzed and interpreted separately, rather than treated as equivalent comparators.

Although most primary studies were exclusive to a single SR, a considerable proportion were duplicated across reviews. This overlap, identified through the GROOVE tool, reinforces the importance of broader, more explicit, and better-justified search strategies in future reviews, as well as transparent management of overlapping primary studies. Future SRs should register their protocols in platforms such as PROSPERO, report deviations from the protocol, update searches periodically, and perform sensitivity analyses when sufficient data are available.

Finally, future RCTs should use adequate sample sizes, pre-specified outcomes, appropriate statistical methods such as intention-to-treat analysis, and standardized reporting of exercise prescription, including frequency, intensity, time, type, progression, supervision, and adherence. Addressing these methodological limitations will enable the generation of more robust evidence to determine the role of specific exercise modalities in the management of BCRL and to support evidence-based clinical recommendations.

### 4.4. Strengths and Limitations

This overview has several limitations that should be considered when interpreting the findings. It was limited to SRs published in English, Spanish, or Portuguese, which may have excluded relevant evidence published in other languages. In addition, because this study was designed as an overview of SRs, we did not conduct an independent search for primary RCTs outside the included reviews, in accordance with Cochrane guidance for overviews. Therefore, although the SR search was updated to March 2025, the comprehensiveness of the trial-level evidence depends on the coverage of the included SRs, and relevant RCTs not included in any eligible review may not have been captured.

The limited number of available studies for each comparison and outcome also prevented the performance of sensitivity analyses, subgroup analyses, and formal publication bias assessments. In particular, publication bias or small-study effects could not be formally assessed because fewer than 10 RCTs contributed to each meta-analysis. Therefore, the presence of publication bias cannot be ruled out and should be considered when interpreting the certainty and comprehensiveness of the evidence.

Another important limitation relates to the methodological quality of the included reviews. All included SRs were judged to be at high overall risk of bias using ROBIS, which limits confidence in the existing review-level literature and justifies caution when interpreting prior pooled conclusions. However, this does not invalidate the overview design; rather, it highlights the need to critically contextualize the evidence base and avoid relying exclusively on the conclusions of the included reviews. Accordingly, as pre-specified in the protocol, eligible and non-duplicated RCT outcome data contained within the included reviews were re-analyzed by comparison and outcome of interest.

Some included SRs had a broader scope, including non-randomized designs, single-group studies, or populations beyond those with established BCRL. Nevertheless, this potential source of contamination was mitigated by extracting and re-analyzing only eligible RCT data corresponding to women with established BCRL and pre-specified outcomes. Therefore, non-RCT evidence and non-eligible populations were not incorporated into the outcome synthesis.

Clinical and methodological heterogeneity should also be considered when interpreting the findings. The main sources of heterogeneity included differences in exercise modality, intervention dose and supervision, comparator type, follow-up time, BCRL measurement methods, outcome definitions, and risk of bias across RCTs. These factors limited the feasibility of formal subgroup or sensitivity analyses and support the need to interpret the findings according to exercise modality, comparator, outcome, and follow-up time.

Despite these limitations, this overview also presents important strengths. It represents the first overview to compile and critically analyze evidence drawn exclusively from SRs based on RCTs evaluating the effects of physical exercise programs in patients with BCRL. The applied methodology was rigorous, with a protocol registered in PROSPERO and guided by the Cochrane Handbook and the PRIOR statement, and internationally recognized tools (ROBIS, RoB 2.0, GRADE, and GROOVE) were used, enabling a comprehensive evaluation of methodological quality, CoE, and degree of overlap among studies. Finally, the clinical and practical orientation of the selected outcomes reinforces the applicability of the findings for healthcare professionals and decision-makers involved in the management of BCRL.

## 5. Conclusions

The effectiveness of exercise-based interventions in women with established BCRL remains uncertain for most modalities and outcomes. Among the modalities evaluated, supervised weightlifting probably reduces the risk of long-term lymphedema volume increase (moderate CoE), whereas aquatic exercise may improve selected short-term shoulder range of motion outcomes (low CoE). However, these findings should be interpreted with exercise modality, comparator, outcome, and follow-up time in mind, rather than as evidence of a common effect across all physical exercise programs.

For other exercise modalities, including resistance exercise, yoga, Pilates, and multicomponent programs, the evidence remains very uncertain for most patient-important outcomes, including QoL, pain, grip strength, and upper-limb function. Although no adverse events were reported in the included studies, the absence of reported harm should not be interpreted as definitive evidence of safety or as sufficient justification for widespread implementation.

Considering the available evidence, the decision to use exercise-based interventions should be made jointly by the treating clinician and the patient, considering the specific exercise modality, clinical objective, functional status, comorbidities, preferences, availability of supervision, and limited CoE. High-quality RCTs evaluating patient-important outcomes, using standardized BCRL assessment methods, and reporting adverse events consistently are needed to confirm these findings and strengthen the evidence base supporting exercise-based interventions in the management of BCRL.

## Figures and Tables

**Table 1:** Characteristics of the SRs included.

Author/Year	Country	Databases Consulted	Search Date	Study Designs Included	Sample Size	Population/Age	No. of Studies Included	Intervention	Components of Intervention	Comparator	Risk of Bias Assessment Tool	Certainty of Evidence	Type of Synthesis
Karki 2009 [67]	Finland	Ovid MEDLINE, CINAHL, CRD (Centre for Reviews and Dissemination), OAIster, PEDro, Cochrane Database of Systematic Reviews, Embase	January 2004 to March 2008	RCTs	NR	Patients with lymphedema after breast cancer treatment/NR	14	Physical therapy for BCRL	Compression bandaging; compression sleeves; manual lymphatic drainage; mechanical or pneumatic lymphatic drainage; therapeutic exercise, including active or passive movement; physical modalities, including laser, electrical stimulation, ultrasound, and heat; any combination of these interventions	No treatment; placebo or sham therapy; alternative physiotherapeutic interventions, such as compression bandaging or manual lymphatic drainage; combination of treatments	Cochrane RoB 1	Van Tulder criteria	Qualitative
Lian 2024 [68]	Ireland	PubMed, Embase, Scopus	Until November 2023	RCTs	NR	Adults with BCRL/>18 years	13	Structured exercise programs	Yoga; resistance exercise; combined resistance and aerobic exercise; aquatic exercise	Self-care or standard care without structured exercise	Cochrane RoB 1	NR	Qualitative
Muñoz-Gómez 2023 [69]	Spain	PubMed, Web of Science, Cochrane Library	Last 10 years, 2010 to 2020	RCTs	606	Breast cancer survivors/mean age: 56.67 years	10	Aquatic therapeutic exercise	Aquatic aerobic exercise; aquatic resistance training; respiratory exercises; joint mobility and stretching; lymphatic self-massage; specific aquatic modalities, such as Ai Chi	Standard care; non-classified interventions such as therapeutic physical exercise, either in water or on land	PEDro; Jadad scale	NR	Qualitative
Rogan 2016 [44]	Switzerland	CINAHL, Cochrane Central Register of Controlled Trials, PEDro	Until January 2016	RCTs; pre–post studies	NR	Patients with breast cancer/NR	32	Therapeutic modalities	Lymphatic drainage; kinesiotape or lymphatic taping; compression bandaging; compression sleeve; intermittent pneumatic compression; exercise	Control intervention; exercise	RoB tool	NR	Quantitative and qualitative
Paramanandam 2014 [70]	United Kingdom	PubMed, EMBASE, PsycINFO, CINAHL, AMED, Cochrane, PEDro, SPORTDiscus, Web of Science	July/August 2012	RCTs	1091	Women with or at risk of BCRL/range: 49 to 57 years	11	Progressive weight-training exercise	Weight training or resistance exercises	No intervention; sham exercise; light exercise; gentle stretching; lower-limb exercises	PEDro	NR	Quantitative and qualitative
Singh 2016 [71]	Australia	CINAHL, Cochrane, EBSCOhost, MEDLINE, PubMed, ProQuest Health and Medical Complete, ProQuest Nursing and Allied Health Source, ScienceDirect, SPORTDiscus	Until January 2015	RCTs; non-RCTs; single-group pre–post studies	NR	Patients with extremity lymphedema related to breast cancer or other cancers/NR	Review 1: 20; Review 2: 3; total: 23	Resistance exercise	Weightlifting; resistance exercise; aerobic exercise; other types of exercise	No exercise; other types of exercise; standard care	EPHPP	NHMRC	Quantitative and qualitative
Hayes 2022 [72]	Australia	Cochrane Library, PubMed, CINAHL, SPORTDiscus via EBSCOhost, EMBASE, ProQuest Health and Medical Complete, ProQuest Nursing and Allied Health Source	Until March 2021	RCTs; non-RCTs; single-group pre–post studies	NR	Patients at risk of, or with, cancer-related lymphedema affecting upper limb after breast cancer or lower limb after other cancers, including melanoma, gynecological, and head and neck cancers/mean age: 55 years; range: 29 to 58 years	Objective 1: 10; Objective 2: 26; total: 36	Exercise	Objective 1: Exercise for prevention of cancer-related lymphedema; Objective 2: exercise for treatment of lymphedema	No exercise; other types of exercise; standard care	EPHPP	GRADE	Quantitative and qualitative
Wanchai 2018 [73]	Thailand	ScienceDirect, PubMed, Scopus, CINAHL	2007 to 2017	RCTs	NR	Patients with BCRL or at risk of developing BCRL/NR	15	Resistance training	Weight training; resistance exercises	No exercise; other types of exercise; standard care	PEDro	NR	Qualitative
Naghibi 2018 [47]	Iran	PubMed, MEDLINE, CINAHL, Google Scholar	Until August 2016	RCTs; non-RCTs	NR	Patients with BCRL/NR	12	Exercise training	Physical training; exercise therapy; physical activity	No exercise	TGCPS	NR	Qualitative

NR: Not reported; EPHPP: Effective Public Health Practice Project Quality Assessment Tool; NHMRC: National Health and Medical Research Council; BCRL: breast cancer-related lymphedema; TGCPS: Guide to Community Preventive Services. Note: Table 1 describes the population scope of the included systematic reviews. Some reviews included mixed populations, including both women with established BCRL and at risk of developing BCRL. For this overview’s outcome synthesis, only RCT data corresponding to women with established BCRL were extracted and re-analyzed when these data were reported separately or could be clearly identified.

**Table 2:** Evaluation of SR risk of bias according to ROBIS.

Review	Phase 2				Phase 3
	1. STUDY ELIGIBILITY CRITERIA	2. IDENTIFICATION AND SELECTION OF STUDIES	3. DATA COLLECTION AND STUDY APPRAISAL	4. SYNTHESIS AND FINDINGS	RISK OF BIAS IN THE REVIEW
Karki 2009 [67]	☹	☹	☹	☹	☹
Lian 2024 [68]	☹	☹	☹	☹	☹
Muñoz-Gómez 2023 [69]	☹	☹	☹	☹	☹
Rogan 2016 [44]	☹	☹	☹	☹	☹
Paramanandam 2014 [70]	☺	☹	☹	☹	☹
Singh 2016 [71]	☹	☹	☺	☹	☹
Hayes 2022 [72]	?	☹	?	☺	☹
Wanchai 2018 [73]	☹	☹	☹	☹	☹
Naghibi 2018 [47]	☺	☹	☹	☹	☹

Note: ROBIS, Risk of Bias in Systematic Reviews. ☺ = low concern or low risk of bias; ☹ = high concern or high risk of bias; ? = unclear concern or unclear risk of bias.

## Data Availability

The raw data supporting the conclusions of this article will be made available by the authors on request.

## Supplementary Materials

The following supporting information can be downloaded at: https://www.mdpi.com/article/10.3390/jcm15135001/s1. Supplement S1: PRIOR verification list of guidelines for reporting summaries of reviews of health interventions; Supplement S2: Volumetric definitions and formulae; Supplement S3: Search strategy for the Medline/PubMed database; Supplement S4: Search strategy used on Lilacs; Supplement S5: Search strategy used on PEDro; Supplement S6: Search strategy used on Cochrane Library; Supplement S7: Search strategy used on Embase (Ovid); Supplement S8: Excluded systematic reviews and rationale for exclusion; Supplement S9: Characteristics of the excluded RCTs; Supplement S10: Additional information on the characteristics of the included systematic reviews and meta-analyses; Supplement S11: Overall results of CCA; Supplement S12: Risk of bias evaluation in the RCTs; Supplement S13: Summary of effect and certainty of evidence estimators for primary results; Supplement S14: Summary of effect and certainty of evidence estimators for secondary results.

## Abbreviations

The following abbreviations are used in this manuscript:

BCRL	Breast cancer-related lymphedema
QoL	Quality of life
AE	Aerobic exercise
RE	Resistance exercise
CoE	Certainty of evidence
GRADE	Grading of Recommendations Assessment, Development and Evaluation
PRIOR Statement	Preferred Reporting Items for Overviews of Reviews
SRs	Systematic reviews
RCTs	Randomized controlled trials
EORTC QLQ-C30	European Organisation for Research and Treatment of Cancer Quality of Life
NRS	Numeric rating scale
VAS	Visual analogue scale
DASH	Disabilities of the Arm, Shoulder and Hand
CCA	Corrected Covered Area
RR	Relative risk
CI	Confidence interval
MD	Mean difference
SMD	Standardized mean difference
non-RCTs	Non-randomized controlled trials
GROOVE	Graphical Representation of Overlap for Overviews
LV	Lymphedema volume
VR	Volume reduction
AEs	Adverse events
ROM	Range of motion
