# Supplementary material for: Effectiveness of Physical Exercise Programs in Reducing Secondary Lymphedema Associated with Breast Cancer: An Overview of Systematic Reviews
Source: J Clin Med. 2026 Jun 26;15(13):5001. doi: 10.3390/jcm15135001 (PMC13362082; doi:10.3390/jcm15135001)
Supplement: Supplementary file 1 [file jcm-15-05001-s001.zip › jcm-4355888-supplementary.pdf]

Supplemental material

Effectiveness of physical exercise programs in reducing secondary lymphedema associated with breast cancer: An overview of systematic reviews.

Table of contents

Effectiveness of physical exercise programs in reducing secondary lymphedema associated with breast cancer: An overview of systematic reviews. 1

Supplement 1: PRIOR Verification List of Guidelines for Reporting Summaries of Reviews of Health Interventions........ 3

Supplement 2: Volumetric definitions and Formulae 7

Supplement 3: Search strategy for the Medline/PubMed database........ 9

Supplement 4: Search strategy used on Lilacs: 10

Supplement 5: Search strategy used on PEDro 11

Supplement 6: Search strategy used on Cochrane Library........ 12

Supplement 7: Search strategy used on Embase (Ovid) 13

Supplement 8: Excluded systematic reviews and rationale for exclusion. 14

Supplement 12: Characteristics of the excluded RCTs 22

Supplement 10: Additional information for the characteristics of the systematic reviews and metanalyses included........ 29

Supplement 11: Overall results of CCA 30

Supplement 12: Evaluation of risk of bias in the RCTs 31

Supplement 13: Summary of effect and certainty of evidence estimators for primary results........ 39

Supplement 14: Summary of effect and certainty of evidence estimators for secondary results 41

Supplement S1: PRIOR Verification List of Guidelines for Reporting Summaries of Reviews of Health Interventions

**Section** Topic	#	**Item**	**Location reported**
**TITLE**			
Title	1	Identify the report as an overview of reviews.	1-2
**ABSTRACT**			
Abstract	2	Provide a comprehensive and accurate summary of the purpose, methods, and results of the overview of reviews.	48-72
**INTRODUCTION**			
Rationale	3	Describe the rationale for conducting the overview of reviews in the context of existing knowledge.	96-130
Objectives	4	Provide an explicit statement of the objective(s) or question(s) addressed by the overview of reviews.	131-134
**METHODS**			
Eligibility criteria	5a	Specify the inclusion and exclusion criteria for the overview of reviews. If supplemental primary studies were included, this should be stated, with a rationale.	145-197
	5b	Specify the definition of 'systematic review' as used in the inclusion criteria for the overview of reviews.	145-159
Information sources	6	Specify all databases, registers, websites, organizations, reference lists, and other sources searched or consulted to identify systematic reviews and supplemental primary studies (if included). Specify the date when each source was last searched or consulted.	199-208
Search strategy	7	Present the full search strategies for all databases, registers and websites, such that they could be reproduced. Describe any search filters and limits applied.	Supplements 3-7
Selection process	8a	Describe the methods used to decide whether a systematic review or supplemental primary study (if included) met the inclusion criteria of the overview of reviews.	210-217
	8b	Describe how overlap in the populations, interventions, comparators, and/or outcomes of systematic reviews was identified and managed during study selection.	210-217
Data collection process	9a	Describe the methods used to collect data from reports.	210-232
	9b	If applicable, describe the methods used to identify and manage primary study overlap at the level of the comparison and outcome during data collection. For each outcome, specify the method used to illustrate and/or quantify the degree of primary study overlap across systematic reviews.	210-232
	9c	If applicable, specify the methods used to manage discrepant data across systematic reviews during data collection.	210-232

Data items	10	List and define all variables and outcomes for which data were sought. Describe any assumptions made and/or measures taken to identify and clarify missing or unclear information.	210-232
Risk of bias assessment	11a	Describe the methods used to *assess* risk of bias or methodological quality of the included systematic reviews.	234-260
	11b	Describe the methods used to *collect* data on (from the systematic reviews) and/or *assess* the risk of bias of the primary studies included in the systematic reviews. Provide a justification for instances where flawed, incomplete, or missing assessments are identified but not re-assessed.	234-260
	11c	Describe the methods used to *assess* the risk of bias of supplemental primary studies (if included).	234-260
Synthesis methods	12a	Describe the methods used to summarize or synthesize results and provide a rationale for the choice(s).	297-326
	12b	Describe any methods used to explore possible causes of heterogeneity among results.	297-326
	12c	Describe any sensitivity analyses conducted to assess the robustness of the synthesized results.	297-326
Reporting bias assessment	13	Describe the methods used to *collect* data on (from the systematic reviews) and/or *assess* the risk of bias due to missing results in a summary or synthesis (arising from reporting biases at the levels of the systematic reviews, primary studies, and supplemental primary studies, if included).	297-326
Certainty assessment	14	Describe the methods used to *collect* data on (from the systematic reviews) and/or *assess* certainty (or confidence) in the body of evidence for an outcome.	267-295
**RESULTS**			
Systematic review and supplemental primary study selection	15a	Describe the results of the search and selection process, including the number of records screened, assessed for eligibility, and included in the overview of reviews, ideally with a flow diagram.	328-521
	15b	Provide a list of studies that might appear to meet the inclusion criteria, but were excluded, with the main reason for exclusion.	328-521
**Section** Topic	**#**	**Item**	**Location reported**
Characteristics of systematic reviews and supplemental primary studies	16	Cite each included systematic review and supplemental primary study (if included) and present its characteristics.	328-521
Primary study overlap	17	Describe the extent of primary study overlap across the included systematic reviews.	328-521
Risk of bias in systematic reviews, primary studies, and supplemental primary studies	18a	Present assessments of risk of bias or methodological quality for each included systematic review.	328-521
	18b	Present assessments (*collected* from systematic reviews or *assessed* anew) of the risk of bias of the primary studies included in the systematic reviews.	328-521
	18c	Present assessments of the risk of bias of supplemental primary studies (if included).	328-521

Summary or synthesis of results	19a	For all outcomes, summarize the evidence from the systematic reviews and supplemental primary studies (if included). If meta-analyses were done, present for each the summary estimate and its precision and measures of statistical heterogeneity. If comparing groups, describe the direction of the effect.	328-521
	19b	If meta-analyses were done, present results of all investigations of possible causes of heterogeneity.	328-521
	19c	If meta-analyses were done, present results of all sensitivity analyses conducted to assess the robustness of synthesized results.	328-521
Reporting biases	20	Present assessments (*collected* from systematic reviews and/or *assessed* anew) of the risk of bias due to missing primary studies, analyses, or results in a summary or synthesis (arising from reporting biases at the levels of the systematic reviews, primary studies, and supplemental primary studies, if included) for each summary or synthesis assessed.	328-521
Certainty of evidence	21	Present assessments (*collected* or *assessed* anew) of certainty (or confidence) in the body of evidence for each outcome.	328-521
**DISCUSSION**			
Discussion	22a	Summarize the main findings, including any discrepancies in findings across the included systematic reviews and supplemental primary studies (if included).	523-634
	22b	Provide a general interpretation of the results in the context of other evidence.	523-634
	22c	Discuss any limitations of the evidence from systematic reviews, their primary studies, and supplemental primary studies (if included) included in the overview of reviews. Discuss any limitations of the overview of reviews methods used.	523-634
	22d	Discuss implications for practice, policy, and future research (both systematic reviews and primary research). Consider the relevance of the findings to the end users of the overview of reviews, e.g., healthcare providers, policymakers, patients, among others.	523-634
**OTHER INFORMATION**			
Registration and protocol	23a	Provide registration information for the overview of reviews, including register name and registration number, or state that the overview of reviews was not registered.	72
	23b	Indicate where the overview of reviews protocol can be accessed, or state that a protocol was not prepared.	137-143
	23c	Describe and explain any amendments to information provided at registration or in the protocol. Indicate the stage of the overview of reviews at which amendments were made.	141-147-
Support	24	Describe sources of financial or non-financial support for the overview of reviews, and the role of the funders or sponsors in the overview of reviews.	1024
Competing interests	25	Declare any competing interests of the overview of reviews' authors.	1026
Author	26a	Provide contact information for the corresponding author.	35

information	26b	Describe the contributions of individual authors and identify the guarantor of the overview of reviews.	666-677
Availability of data and other materials	27	Report which of the following are available, where they can be found, and under which conditions they may be accessed: template data collection forms; data collected from included systematic reviews and supplemental primary studies; analytic code; any other materials used in the overview of reviews.	693

Supplement S2: Volumetric definitions and Formulae

**Volumetric Outcome**	**Definition / Explanation**	**Formula**	**Other terms for this outcome in the included studies**
Lymphedema Volume	The excess volume in the limb. The volume is measured in milliliters (mL). It is called 'lymphedema volume' because it is the amount of the limb volume that is attributed to lymphedema. Lymphedema volume is NOT the total volume of the limb. To calculate the lymphedema volume, you have to compare the affected limb to the unaffected limb by subtracting. In this review, we are interested in the lymphedema volume (or excess volume) that remains in the limb after treatment.	Post-treatment total volume of the affected arm minus posttreatment total volume of the unaffected arm.	**Lymphedema volume**<br><br>has also been called absolute lymphedema volume, post-intervention volume (McNeely 2004), and excess limb volume (Williams 2002).
Volume Reduction	An estimate of how much the limb has been reduced (in ml) presumably from the treatment.	Lymphedema volume at baseline minus the lymphedema volume after treatment.<br><br>OR<br><br>Excess volume before treatment minus the excess volume after treatment.	**Volume reduction**<br><br>has also been called the mean lymphedema volume reduction (Johansson 1998; Johansson 1999) and mean change lymphedema volume (McNeely 2004).
Per cent Reduction	The decrease in excess volume relative to the amount of excess volume at baseline. Both the lymphedema volume and the volume reduction are considered absolute values not relative values. However, when absolute values are used,	$\frac{\text{Difference Test A} - \text{Difference Test B}}{\text{Difference Test A}} \times 100$	**Per cent Reduction**<br><br>has also been called the percentage lymphedema reduction (Johansson 1998; Johansson 1999), per cent

	a person with a large excess limb volume might get a 2% reduction, but the amount can look large because the beginning volume was large. By contrast, a person with a small beginning volume can get a 30% reduction, and it can look small in absolute terms. Thus, it is valuable to have a third way to think about lymphedema outcomes, and that is to look at the per cent change because that is a relative value.	Where difference is the affected arm volume minus the unaffected arm volume (McNeely 2004)<br><br>Another way to think of per cent reduction is this formula:<br><br>Excess volume at baseline – Excess volume post treatment____________________________________ x 100<br><br>Excess volume at baseline	change, per cent reduction in lymphedema volume (McNeely 2004), percentage change in excess limb volume (Sitzia 2002).

From: Ezzo J, Manheimer E, McNeely ML, Howell DM, Weiss R, Johansson KI, Bao T, Bily L, Tuppo CM, Williams AF, Karadibak D. Manual lymphatic drainage for lymphedema following breast cancer treatment. Cochrane Database of Systematic Reviews 2015, Issue 5. Art. No.: CD003475. DOI: 10.1002/14651858.CD003475.

Supplement S3: Search strategy for the Medline/PubMed database

Data base	Search terms
Medline/PubMed	#1 "Breast Cancer Lymphedema"[Mesh]<br>#2 "Breast Cancer Lymphedema"<br>#3 Lymphedema, Breast Cancer<br>#4 Breast Cancer Treatment-Related Lymphedema<br>#5 Postmastectomy Lymphedema<br>#6 lymphoedema<br>#7 upper-limb lymphedema<br>#8 breast cancer associated lymphoedema<br>#9 Lymphedema<br>#10 or/1-9<br>#11 Physical Therapy Techniques<br>#12 Physical Therapy Specialty<br>#13 EXERCISE THERAPY<br>#14 EXERCISE MOVEMENT TECHNIQUES<br>#15 EXERCISE<br>#16 REHABILITATION<br>#17 Physical fitness<br>#18 "Exercise"[Mesh]<br>#19 Physical Activity<br>#20 Isometric<br>#21 Aerobic<br>#22 Training<br>#23 "Endurance Training"[Mesh]<br>#24 "Endurance Training"<br>#25 Physiotherapy<br>#26 "Yoga"<br>#27 "Aquatic Therapy"[Mesh]<br>#28 "Aquatic Therapy"<br>#29 Weightlifting<br>#30 Pilates<br>#31 or/11-30<br>#32 search*[Title/Abstract]<br>#33 meta analysis[Publication Type]<br>#34 meta analysis[Title/Abstract]<br>#35 meta analysis[MeSH Terms]<br>#36 review[Publication Type]<br>#37 diagnosis[MeSH Subheading]<br>#38 associated[Title/Abstract]<br>#39 or/32-38<br>#40 and/10, 31, 39

Supplement S4: Search strategy used on Lilacs:

Data base	Search terms
Lilacs	#1 linfedema del cáncer de mama<br>#2 linfedema posmastectomía<br>#3 linfedema del brazo relacionado con el cáncer de mama<br>#4 or/1-3<br>#5 fisioterapia (técnicas)<br>#6 técnicas de fisioterapia<br>#7 especialidad de terapia física<br>#8 ejercicio de rehabilitación<br>#9 método pilates<br>#10 técnicas de ejercicios con movimiento<br>#11 ejercicio físico<br>#12 resistencia física<br>#13 ejercicio aeróbico<br>#14 ejercicio isométrico<br>#15 entrenamiento de resistencia<br>#16 yoga<br>#17 terapia de ejercicios acuáticos<br>#18 or/5-17<br>#19 Filter: (type_of_study:("systematic_reviews")<br>#20 and/18,19

Supplement S5: Search strategy used on PEDro

Advanced Search:

Abstract & Title: Breast Cancer Lymphedema Exercise

Therapy: -

Problem: -

Body Part: upper arm, shoulder or shoulder girdle

Subdiscipline: -

Topic: -

Method: systematic review

Autor/Association: -

Title Only: -

Source: -

Published Since: -

New records added since: -

Score at least: -

When Searching: Match all search terms (AND)

Supplement S6: Search strategy used on Cochrane Library

Data base	Search terms
Cochrane Library	#1 MeSH descriptor: [Breast Cancer Lymphedema] explode all trees<br>#2 (Breast Cancer Lymphedema):kw<br>#3 (lymphoedema):kw<br>#4 (Postmastectomy Lymphedema):kw<br>#5 (Breast Cancer Treatment-Related Lymphedema):kw<br>#6 (Physical Therapy Modalities):kw<br>#7 MeSH descriptor: [Exercise] explode all trees<br>#8 MeSH descriptor: [Rehabilitation] explode all trees<br>#9 MeSH descriptor: [Physical Fitness] explode all trees<br>#10 MeSH descriptor: [Endurance Training] explode all trees<br>#11 (Endurance Training):kw<br>#12 MeSH descriptor: [Yoga] explode all trees<br>#13 MeSH descriptor: [Aquatic Therapy] explode all trees<br>#14 MeSH descriptor: [Exercise Movement Techniques] explode all trees<br>#15 ("weightlifting"):kw<br>#16 (Pilates):kw<br>#17 ("aerobic"):kw<br>#18 ("training"):kw<br>#19 (Physical Therapy Techniques):kw<br>#20 (Physical Therapy Specialty):kw<br>#21 or/#1-5<br>#22 or/#6-20<br>#23 #21 and #22

Supplement S7: Search strategy used on Embase (Ovid)

Data base	Search terms
EMBASE (Ovid)	#1 'breast cancer lymphedema'/exp<br>#2 'breast cancer treatment-related lymphedema'<br>#3 'postmastectomy lymphedema'<br>#4 'upper limb lymphedema'/exp<br>#5 'breast cancer associated lymphoedema'<br>#6 'lymphedema'/exp<br>#7 or/1-6<br>#8 'physical therapy techniques'/exp<br>#9 'physical therapy specialty'/exp<br>#10 'exercise therapy'/exp<br>#11 'exercise movement techniques'/exp<br>#12 'rehabilitation'/exp<br>#13 'physical activity'/exp<br>#14 'endurance training'/exp<br>#15 'physiotherapy'/exp<br>#16 'yoga'/exp<br>#17 'aquatic therapy'/exp<br>#18 'pilates'/exp<br>#19 'weight training'/exp<br>#20 or/8-19<br>#21 and/7, 20<br>#22 [systematic review]/lim<br>#23 [meta analysis]/lim<br>#24 or/22-23<br>#25 and/21, 24

Supplement S8: Excluded systematic reviews and rationale for exclusion.

N°	ID	Journal	Study	Exclusion Reason
1	Shamoun 2023 [1]	Asian Pac J Cancer Prev, 24 (7), 2225-2238	Complete Decongestive Therapy Effect on Breast Cancer Related to Lymphedema: A Systemic Review and Meta-Analysis of Randomized Controlled Trials	Intervention
2	Qiao 2023 [2]	Cancer Nursing, 46 (2), 159-166	Effect of Manual Lymphatic Drainage on Breast Cancer–Related Postmastectomy Lymphedema	Intervention
3	Mahmood 2022 [3]	BMC Cancer, 22:937	Clinical application of low-level laser therapy (Photo-biomodulation therapy) in the management of breast cancer-related lymphedema: a systematic revie	Intervention
4	Bagatini 2018 [4]	Acta Fisiatr. 25(4):205-210.	Use of shockwave therapy for the treatment of lymphedema associated to breast cancer: a systematic review	Intervention
5	Balcombe 2017 [5]	Oedema. 2017;6–14.	Approaches to the application and removal of compression therapy: A literature review	Intervention/Population/Design
6	Baxter 2017 [6]	BMC Cancer (2017) 17:833	Low level laser therapy (Photobiomodulation therapy) for breast cancer-related lymphedema: a systematic review	Intervention
7	Rangon 2022 [7]	Archives of Physical Medicine and Rehabilitation 2021;000: 1-11	Effects of Complex Physical Therapy and Multimodal Approaches on Lymphedema Secondary to Breast Cancer: A Systematic Review and Meta-analysis of Randomized Controlled Trials	Intervention/
8	Braz 2009 [8]	Rev latino-americana de enfermagem; 17(5)	Tratamientos fisioterapéuticos para el linfedema después de la cirugía de cáncer de seno: una revisión de literatura	Design
9	Cendron 2015 [9]	Revista Brasileira de Cancerologia 2015; 61(1): 49-58	Complex Decongestive Physiotherapy Associated Compression Therapy in the Treatment of Secondary Lymphedema in Breast Cancer: A Systematic Review	Intervention

10	Chan 2010 [10]	Journal of Advanced Nursing 66(9), 1902–1914.	Effectiveness of exercise programmes on shoulder mobility and lymphoedema after axillary lymph node dissection for breast cancer: systematic review	Population
11	Cheifetz 2010 [11]	Can Fam Physician 2010; 56:127784	Management of secondary lymphedema related to breast cancer	Design
12	De Groef 2015 [12]	ARCHIVES OF PHYSICAL MEDICINE AND REHABILITATION (2015)	Effectiveness of Postoperative Physical Therapy for Upper Limb Impairments Following Breast Cancer Treatment: A Systematic Review	Population
13	Donahue 2023 [13]	Breast Cancer Research and Treatment (2023) 200:1–14	Advances in the prevention and treatment of breast cancer-related lymphedema	Design
14	Gatt 2017 [14]	European Journal of Cancer Care, 2017	A meta-analysis of the effectiveness and safety of kinesiology taping in the management of cancer-related lymphoedema	Intervention
15	Gerland 2021 [15]	Breast Care 2021; 16:657–663	Resistance Exercise for Breast Cancer Patients? Evidence from the Last Decade	Design
16	Pereira de Godoy 2022 [16]	Literature Review. Cureus 14(1): e21263.	Mechanical Lymphatic Drainage (RAGodoy®): Literature Review	Design/Intervention
17	Gomide 2007 [17]	Int J Clin Pract, June 2007, 61, 6, 972–982	Morbidity after breast cancer treatment and physiotherapeutic performance	Design
18	Hill 2024 [18]	Disability and Rehabilitation https://doi.org/10.1080/09638288.2023.2231842	The effectiveness and safety of heat/cold therapy in adults with lymphoedema: systematic review	Intervention
19	Huang 2013 [19]	World Journal of Surgical Oncology 2013, 11:15	Effects of manual lymphatic drainage on breast cancer-related lymphedema: a systematic review and meta-analysis of randomized controlled trials	Intervention
20	Jang 2020 [20]	Medicine (2020) 99:50	Acupuncture as an adjuvant therapy for management of treatment-related symptoms in breast cancer patients Systematic review and meta-analysis (PRISMA-compliant)	Intervention/Population
21	Kligman 2004 [21]	Support Care Cancer (2004) 12:421–431	The treatment of lymphedema related to breast cancer: a systematic review and evidence summary	Design

22	Kasawara 2018 [22]	PHYSIOTHERAPY THEORY AND PRACTICE https://doi.org/10.1080/09593985.2017.1419522	Effects of Kinesio Taping on breast cancer-related lymphedema: A meta-analysis in clinical trials	Intervention
23	Lasinski 2012 [23]	the American Academy of Physical Medicine and Rehabilitation Vol. 4, 580-601, August 2012	A Systematic Review of the Evidence for Complete Decongestive Therapy in the Treatment of Lymphedema From 2004 to 2011	Intervention/Design
24	Lin 2023 [24]	Nursing Open. 2023; 10:2030–2043.	Effect of exercise on rehabilitation of breast cancer surgery patients: A systematic review and meta-analysis of randomized controlled trials	Population
25	Maccarone 2023 [25]	Journal of Vascular Surgery: Venous and Lymphatic Disorders	Water-based exercise for upper and lower limb lymphoedema treatment	Design
26	Ezzo 2015 [26]	Cochrane Database of Systematic Reviews 2015, Issue 5. Art. No.: CD003475.	Manual lymphatic drainage for lymphedema following breast cancer treatment	Intervention
27	Marotta 2023 [27]	European Journal of Physical and Rehabilitation Medicine 2023 Feb 27	Efficacy of kinesio taping on upper limb volume reduction in patients with breast cancer-related lymphedema: a systematic review of randomized controlled trials	Intervention
28	Merchant 2015 [28]	The Breast Journal, Volume 21 Number 3, 2015 276–284	Prevention and Management of Lymphedema after Breast Cancer Treatment	Design
29	Miccinilli 2020 [29]	Lymphology 53 (2020) 118-135	The effectiveness of extracorporeal shock wave therapy on breast cancer-related lymphedema: a literature review	Intervention
30	Michelotti 2019 [30]	The Breast 44 (2019) 15e23	Tackling the diversity of breast cancer related lymphedema: Perspectives on diagnosis, risk assessment, and clinical management	Design/ Intervention
31	Morris 2013 [31]	Physiotherapy Theory and Practice, 29:4, 259-270	The clinical effects of Kinesio® Tex taping: A systematic review	Intervention
32	Müller 2018 [32]	Quality of Life Research (2018) 27:1403–1414	Manual lymphatic drainage and quality of life in patients with lymphoedema and mixed oedema: a systematic review of randomised controlled trials	Intervention
33	Mur-Gimeno 2022 [33]	EurJCancerCare.2021;1–16.	Systematic review of the effect of aquatic therapeutic exercise in breast cancer survivors	Population

34	Omar 2012 [34]	Support Care Cancer (2012) 20:2977–2984	A systematic review of the effect of low-level laser therapy in the management of breast cancer-related lymphedema	Intervention
35	Oremus 2012 [35]	BMC Cancer 2012, 12:6	Systematic review: conservative treatments for secondary lymphedema	Intervention
36	Pan 2014 [36]	Int J Clin Oncol	Massage interventions and treatment-related side effects of breast cancer: a systematic review and meta-analysis	Intervention
37	Perdomo 2023 [37]	Journal of Cancer Survivorship (2023) 17:384–398	Patient education for breast cancer–related lymphedema: a systematic review	Intervention
38	Robijns 2017 [38]	Lasers Med Sci (2017) 32:229–242	The use of low-level light therapy in supportive care for patients with breast cancer: review of the literature	Intervention
39	Shao 2017 [39]	European Journal of Cancer Care, 2016	Manual lymphatic drainage for breast cancer-related lymphoedema	Intervention
40	Rafn 2024 [40]	The Lancet; 67 2024	Examining the efficacy of treatments for arm lymphedema in breast cancer survivors: an overview of systematic reviews with meta-analyses	Design
41	Smykla 2013 [41]	BioMed Research International Volume 2013, Article ID 767106, 7 pages	Effect of Kinesiology Taping on Breast Cancer-Related Lymphedema: A Randomized Single-Blind Controlled Pilot Study	Design/ Intervention
42	Tatham 2013 [42]	Physiotherapy Canada 2013; 65(4);321–330	The Efficacy of Exercise Therapy in Reducing Shoulder Pain Related to Breast Cancer: A Systematic Review	Population
43	Thompson 2021 [43]	Journal of Cancer Survivorship (2021) 15:244–258	Manual lymphatic drainage treatment for lymphedema: a systematic review of the literature	Intervention
44	Academy of Oncologic Physical Therapy 2020 [44]	Academy of Oncologic Physical Therapy CSM 2020 Platform and Poster Presentations	Impact of physical activity on lower extremity lymphedema in patients with gynecological cancer: systematic review	Design
45	Tsai 2021 [45]	Clin. Med. 2021, 10, 5970	Extracorporeal Shock Wave Therapy Combined with Complex Decongestive Therapy in Patients with	Intervention

			Breast Cancer-Related Lymphedema: A Systemic Review and Meta-Analysis	
46	Wang 2022 [46]	Lasers in Medical Science (2022) 37:1389–1413	The effectiveness and safety of low-level laser therapy on breast cancer–related lymphedema: An overview and update of systematic reviews	Design /Intervention
47	Xing 2023 [47]	Eur. J. Gynaecol. Oncol. 2023 vol.44(1), 1-16	Effectiveness of manual lymphatic drainage for breast cancer-related lymphoedema: an overview of systematic reviews and meta-analyses	Design
48	Devoogdt 2010 [48]	European Journal of Obstetrics & Gynecology and Reproductive Biology 149 (2010) 3–9	Different physical treatment modalities for lymphoedema developing after axillary lymph node dissection for breast cancer: A review	Intervention
49	Finnane 2015 [49]	Am. J. Phys. Med. Rehabil. & Vol. 94, No. 6, June 2015	Review of the Evidence of Lymphedema Treatment Effect	Design
50	Ding 2020 [50]	ONCOLOGY NURSING FORUM SEPTEMBER 2020, VOL. 47, NO.	Prospective Surveillance and Risk Reduction of Cancer Treatment–Related Lymphedema: Systematic Review and Meta-Analysis	Population
51	Hayes 2020 [51]	Current Breast Cancer Reports (2020) 12:193–201	Do Women with Breast Cancer–related Lymphoedema Need to Wear Compression While Exercising? Results from a Systematic Review and Meta-analysis	Comparator
52	Kwan 2011 [52]	J Cancer Surviv (2011) 5:320–336	Exercise in patients with lymphedema: a systematic review of the contemporary literature	Design
53	Liang 2020 [53]	Medicine (2020) 99:49	Manual lymphatic drainage for lymphedema in patients after breast cancer surgery A systematic review and meta-analysis of randomized controlled trials	Intervention
54	Megens 1998 [54]	Physical Therapy. Volume 78. Number 12. December 1998	Physical Therapist Management of Lymphedema Following Treatment for Breast Cancer: A Critical Review of Its Effectiveness	Intervention
55	Moseley 2007 [55]	Annals of Oncology Volume 18|No. 4|April 2007	A systematic review of common conservative therapies for arm lymphoedema secondary to breast cancer treatment	Intervention/Comparator

56	Naik 2021 [56]	Journal of Clinical and Diagnostic Research. 2021 May, Vol-15(5): YE01-YE05	Effect of Physiotherapy in the Prevention and Relief of Secondary Lymphoedema in Subjects with Postoperative Breast Cancer- A Systematic Review of Randomised Controlled Trials	Intervention/Comparator
57	Panchik 2019 [57]	Journal of Reconstructive Microsurgery 2018	The Effect of Exercise on Breast Cancer–Related Lymphedema: What the Lymphatic Surgeon Needs to Know	Comparator
58	Ridner 2012 [58]	Nursing Research July/August 2012 Vol 61, No 4, 291–299	Self-Management of Lymphedema A Systematic Review of the Literature From 2004 to 2011	Design/Intervention/Comparator
59	Romesberg 2017 [59]	Journal of Women's Health Physical Therapy 2017	The Effects of Resistance Exercises on Secondary Lymphedema Due to Treatment of Breast Cancer: A Review of Current Literature	Design/Comparator
60	Saraswathi 2021 [60]	International Journal of Yoga 2021	Managing Lymphedema, Increasing Range of Motion, and Quality of Life through Yoga Therapy among Breast Cancer Survivors: A Systematic Review	Population/Comparator
61	Wanchai 2020 [61]	Journal of Health Research Vol. 34 No. 5, 2020 pp. 409-418	The effects of yoga on breast-cancer-related lymphedema: a systematic review	Comparator

References:

1. Shamoun, S.; Ahmad, M. Complete decongestive therapy effect on breast cancer related to lymphedema: a systemic review and meta-analysis of randomized controlled trials. Asian Pac. J. Cancer Prev. 2023, 24, 2225–2238
2. Qiao, J.; Yang, L.N.; Kong, Y.H.; Huang, X.; Li, Y.; Bai, D.Q. Effect of manual lymphatic drainage on breast cancer-related postmastectomy lymphedema: a meta-analysis of randomized controlled trials. Cancer Nurs. 2023, 46, 159–166.
3. Mahmood, D.; Ahmad, A.; Sharif, F.; Arslan, S.A. Clinical application of low-level laser therapy (Photo-biomodulation therapy) in the management of breast cancer-related lymphedema: a systematic review. BMC Cancer 2022, 22, 937.
4. Bagatini, O.A.; Bertin, C.; Hong, F.; Guarita, M.L.C.; Shinzato, G.T.; Imamura, M.; Battistella, L.R. Uso da terapia por ondas de choque para o tratamento do linfedema associado ao câncer de mama. Acta Fisiatr. 2018, 25, e0318005.
5. Balcombe, L.; Miller, C.; McGuiness, W. Approaches to the application and removal of compression therapy: a literature review. Br. J. Community Nurs. 2017, 22(Suppl. 10), S6–S14.
6. Baxter, G.D.; Liu, L.; Petrich, S.; Gisselman, A.S.; Chapple, C.; Anders, J.J.; Tumilty, S. Low level laser therapy (Photobio-modulation therapy) for breast cancer-related lymphedema: a systematic review. BMC Cancer 2017, 17, 833.
7. Rangon, F.B.; da Silva, J.; Dibai-Filho, A.V.; Guirro, R.R.J.; Guirro, E.C.O. Effects of complex physical therapy and multimodal approaches on lymphedema secondary to breast cancer: a systematic review and meta-analysis of randomized controlled trials. Arch. Phys. Med. Rehabil. 2022, 103, 353–363
8. Braz, N.F.; Leal, S.; Carrara, H.H.A.; Vieira, K.F.; Homsi, C. Tratamientos fisioterapéuticos para el linfedema después de la cirugía de cáncer de seno: una revisión de literatura. Rev. Latino-Am. Enfermagem 2009, 17, 730–736.
9. Cendron, S.W.; Paiva, L.L.; Darski, C.; Colla, C. Fisioterapia complexa descongestiva associada a terapias de compressão no tratamento do linfedema secundário ao câncer de mama: uma revisão sistemática. Rev. Bras. Cancerol. 2015, 61, 49–58.
10. Chan, D.N.S.; Lui, L.Y.; So, W.K.W. Effectiveness of exercise programmes on shoulder mobility and lymphoedema after axillary lymph node dissection for breast cancer: systematic review. J. Adv. Nurs. 2010, 66, 1902–1914.
11. Cheifetz, O.; Haley, L. Management of secondary lymphedema related to breast cancer. Can. Fam. Physician 2010, 56, 1277–1284.
12. De Groef, A.; Van Kampen, M.; Dieltjens, E.; Christiaens, M.R.; Neven, P.; Geraerts, I.; Devoogdt, N. Effectiveness of post-operative physical therapy for upper-limb impairments after breast cancer treatment: a systematic review. Arch. Phys. Med. Rehabil. 2015, 96, 1140–1153.
13. Donahue, P.M.C.; MacKenzie, A.; Filipovic, A.; Koelmeyer, L. Advances in the prevention and treatment of breast cancer-related lymphedema. Breast Cancer Res. Treat. 2023, 200, 1–14. https://doi.org/10.1007/s10549-023-06897-0
14. Gatt, M.; Willis, S.; Leuschner, S. A meta-analysis of the effectiveness and safety of kinesiology taping in the management of cancer-related lymphoedema. Eur. J. Cancer Care 2017, 26, e12510. https://doi.org/10.1111/ecc.12510
15. Gerland, L.; Baumann, F.T.; Niels, T. Resistance exercise for breast cancer patients? Evidence from the last decade. Breast Care 2021, 16, 657–663. https://doi.org/10.1159/000515227
16. Pereira de Godoy, J.M.; Guerreiro Godoy, M.F.; Pereira de Godoy, H.J. Mechanical lymphatic drainage (RAGodoy®): literature review. Cureus 2022, 14, e21302. https://doi.org/10.7759/cureus.21302
17. Gomide, L.B.; Matheus, J.P.C.; Candido Dos Reis, F.J. Morbidity after breast cancer treatment and physiotherapeutic performance. Int. J. Clin. Pract. 2007, 61, 972–982. https://doi.org/10.1111/j.1742-1241.2006.01124.x
18. Hill, J.E.; Whitaker, J.C.; Sharafi, N.; Hamer, O.; Chohan, A.; Harris, C.; Dawson, S.; Sheridan, B.; Spencer, A.M.; Bee, P.E.; Taylor, G.; Tyson, S.F. The effectiveness and safety of heat/cold therapy in adults with lymphoedema: systematic review. Disabil. Rehabil. 2024, 46, 2184–2195.
19. Huang, T.W.; Tseng, S.H.; Lin, C.C.; Bai, C.H.; Chen, C.S.; Hung, C.S.; Wu, C.H.; Tam, K.W. Effects of manual lymphatic drainage on breast cancer-related lymphedema: a systematic review and meta-analysis of randomized controlled trials. World J. Surg. Oncol. 2013, 11, 15.
20. Jang, S.; Ko, Y.; Sasaki, Y.; Park, S.; Jo, J.; Kang, N.H.; Yoon, J.; Kim, K.H. Acupuncture as an adjuvant therapy for management of treatment-related symptoms in breast cancer patients: systematic review and meta-analysis (PRISMA-compliant). Medicine 2020, 99, e21820.
21. Kligman, L.; Wong, R.K.S.; Johnston, M.; Laetsch, N.S. The treatment of lymphedema related to breast cancer: a systematic review and evidence summary. Support. Care Cancer 2004, 12, 421–431.
22. Kasawara, K.T.; Mapa, J.M.R.; Ferreira, V.; Added, M.A.N.; Shiwa, S.R.; Carvas, N., Jr.; Batista, P.A. Effects of Kinesio Taping on breast cancer-related lymphedema: a meta-analysis in clinical trials. Physiother. Theory Pract. 2018, 34, 337–345.
23. Lasinski, B.B.; Thrift, K.M.K.; Squire, D.C.; Austin, M.K.; Smith, K.M.; Wanchai, A.; Watson, T.; Donahue, P.M.C.; Heckler, C.E.; Armer, J.M. A systematic review of the evidence for complete decongestive therapy in the treatment of lymphedema from 2004 to 2011. PM R 2012, 4, 580–601.
24. Lin, Y.; Chen, Y.; Liu, R.; Cao, B. Effect of exercise on rehabilitation of breast cancer surgery patients: a systematic review and meta-analysis of randomized controlled trials. Nurs. Open 2023, 10, 2030–2043.
25. Maccarone, M.C.; Venturini, E.; Menegatti, E.; Gianesini, S.; Masiero, S. Water-based exercise for upper and lower limb lymphedema treatment. J. Vasc. Surg. Venous Lymphat. Disord. 2023, 11, 201–209.
26. Ezzo, J.; Manheimer, E.; McNeely, M.L.; Howell, D.M.; Weiss, R.; Johansson, K.I.; Bao, T.; Bily, L.; Tuppo, C.M.; Williams, A.F.; Karadibak, D. Manual lymphatic drainage for lymphedema following breast cancer treatment. Cochrane Database Syst. Rev. 2015, 5, CD003475.
27. Marotta, N.; Lippi, L.; Ammendolia, V.; Calafiore, D.; Inzitari, M.T.; Pinto, M.; de Sire, A.; Invernizzi, M. Efficacy of kinesio taping on upper limb volume reduction in patients with breast cancer-related lymphedema: a systematic review of randomized controlled trials. Eur. J. Phys. Rehabil. Med. 2023, 59, 237–247.
28. Merchant, S.J.; Chen, S.L. Prevention and management of lymphedema after breast cancer treatment. Breast J. 2015, 21, 276–284.
29. Miccinilli, S.; Bravi, M.; Maselli, M.; Santacaterina, F.; Morrone, M.; Manco, D.; Brandolini, S.; Sterzi, S.; Bressi, F. The effectiveness of extracorporeal shock wave therapy on breast cancer-related lymphedema: a literature review. Lymphology 2020, 53, 164–180.
30. Michelotti, A.; Invernizzi, M.; Lopez, G.; Lorenzini, D.; Nesa, F.; De Sire, A.; Lippi, L.; Ammendolia, V.; Carda, S.; Picelli, A.; Baima, J.; Cimino, N.; Testa, V.; Fusco, N.; Fontana, F.; Fusco, V. Tackling the diversity of breast cancer related lymphedema: perspectives on diagnosis, risk assessment, and clinical management. Breast 2019, 44, 15–23.
31. Morris, D.; Jones, D.; Ryan, H.; Ryan, C.G. The clinical effects of Kinesio® Tex taping: a systematic review. Physiother.

Theory Pract. 2013, 29, 259–270.
32. Müller, M.; Klingberg, K.; Wertli, M.M.; Carreira, H. Manual lymphatic drainage and quality of life in patients with lymphoedema and mixed oedema: a systematic review of randomised controlled trials. Qual. Life Res. 2018, 27,
33. Mur-Gimeno, E.; Postigo-Martin, P.; Cantarero-Villanueva, I.; Sebio-Garcia, R. Systematic review of the effect of aquatic therapeutic exercise in breast cancer survivors. Eur. J. Cancer Care 2022, 31, e13535.
34. Omar, M.T.A.; Shaheen, A.A.M.; Zafar, H. A systematic review of the effect of low-level laser therapy in the management of breast cancer-related lymphedema. Support. Care Cancer 2012, 20, 2977–2984.
35. Oremus, M.; Dayes, I.; Walker, K.; Raina, P. Systematic review: conservative treatments for secondary lymphedema. BMC Cancer 2012, 12, 6.
36. Pan, Y.Q.; Yang, K.H.; Wang, Y.L.; Zhang, L.P.; Liang, H.Q. Massage interventions and treatment-related side effects of breast cancer: a systematic review and meta-analysis. Int. J. Clin. Oncol. 2014, 19, 829–841.
37. Perdomo, M.; Davies, C.; Levenhagen, K.; Ryans, K.; Gilchrist, L. Patient education for breast cancer-related lymphedema: a systematic review. J. Cancer Surviv. 2023, 17, 384–398.
38. Robijns, J.; Censabella, S.; Bulens, P.; Maes, A.; Mebis, J. The use of low-level light therapy in supportive care for patients with breast cancer: review of the literature. Lasers Med. Sci. 2017, 32, 229–242.
39. Shao, Y.; Zhong, D.S. Manual lymphatic drainage for breast cancer-related lymphoedema. Eur. J. Cancer Care 2017, 26, e12517.
40. Rafn, B.S.; Bodilsen, A.; von Heymann, A.; Lindberg, M.J.; Byllov, S.; Andreasen, T.G.; Johansen, C.; Christiansen, P.; Zachariae, R. Examining the efficacy of treatments for arm lymphedema in breast cancer survivors: an overview of systematic reviews with meta-analyses. EClinicalMedicine 2024, 67, 102397.
41. Smykla, A.; Walewicz, K.; Trybulski, R.; Halski, T.; Kucharzewski, M.; Kucio, C.; Mikusek, W.; Klakla, K.; Taradaj, J. Effect of kinesiology taping on breast cancer-related lymphedema: a randomized single-blind controlled pilot study. Biomed. Res. Int. 2013, 2013, 767106.
42. Tatham, B.; Smith, J.; Cheifetz, O.; Gillespie, J.; Snowden, K.; Temesy, J.; Vandenberk, L. The efficacy of exercise therapy in reducing shoulder pain related to breast cancer: a systematic review. Physiother. Can. 2013, 65, 321–330.
43. Thompson, B.; Gaitatzis, K.; Janse de Jonge, X.; Blackwell, R.; Koelmeyer, L.A. Manual lymphatic drainage treatment for lymphedema: a systematic review of the literature. J. Cancer Surviv. 2021, 15, 244–258.
44. Academy of Oncologic Physical Therapy. Academy of Oncologic Physical Therapy CSM 2020 platform and poster presentations. Rehabil. Oncol. 2020, 38, E11–E31.
45. Tsai, Y.L.; Ting Jie, I.; Chuang, Y.C.; Cheng, Y.Y.; Lee, Y.C. Extracorporeal shock wave therapy combined with complex decongestive therapy in patients with breast cancer-related lymphedema: a systemic review and meta-analysis. J. Clin. Med. 2021, 10, 5640.
46. Wang, Y.; Ge, Y.; Xing, W.; Liu, J.; Wu, J.; Lin, H.; Lu, Y. The effectiveness and safety of low-level laser therapy on breast cancer-related lymphedema: an overview and update of systematic reviews. Lasers Med. Sci. 2022, 37, 1389–1413.
47. Xing, W.; Duan, D.; Ye, C.; Chen, C.; Ge, Y.; Li, Y.; Wang, C.; Zhang, S.; Huang, Y. Effectiveness of manual lymphatic drainage for breast cancer-related lymphoedema: an overview of systematic reviews and meta-analyses. Eur. J. Gynaecol. Oncol. 2023, 44, 1–16.
48. Devoogdt, N.; Van Kampen, M.; Geraerts, I.; Coremans, T.; Christiaens, M.R. Different physical treatment modalities for lymphoedema developing after axillary lymph node dissection for breast cancer: a review. Eur. J. Obstet. Gynecol. Reprod. Biol. 2010, 149, 3–9.
49. Finnane, A.; Janda, M.; Hayes, S.C. Review of the evidence of lymphedema treatment effect. Am. J. Phys. Med. Rehabil. 2015, 94, 483–498.
50. Ding, J.F.; Hasan, B.; Malandris, K.; Farah, M.H.; Manolopoulos, A.; Ginex, P.K.; Salama, L.; Tande, A.J.; Erwin, P.J.; Murad, M.H. Prospective surveillance and risk reduction of cancer treatment-related lymphedema: systematic review and meta-analysis. Oncol. Nurs. Forum 2020, 47, E161–E170.
51. Hayes, S.; Singh, B.; Bloomquist, K.; Johansson, K. Do women with breast cancer-related lymphoedema need to wear compression while exercising? Results from a systematic review and meta-analysis. Curr. Breast Cancer Rep. 2020, 12, 193–201.
52. Kwan, M.L.; Cohn, J.C.; Armer, J.M.; Stewart, B.R.; Cormier, J.N. Exercise in patients with lymphedema: a systematic review of the contemporary literature. J. Cancer Surviv. 2011, 5, 320–336.
53. Liang, M.; Chen, Q.; Peng, K.; Deng, L.; He, L.; Hou, Y.; Zhang, Y.; Guo, J.; Mei, Z.; Li, L. Manual lymphatic drainage for lymphedema in patients after breast cancer surgery: a systematic review and meta-analysis of randomized controlled trials. Medicine 2020, 99, e23192.
54. Megens, A.M.; Harris, S.R. Physical therapist management of lymphedema following treatment for breast cancer: a critical review of its effectiveness. Phys. Ther. 1998, 78, 1302–1311.
55. Moseley, A.L.; Carati, C.J.; Piller, N.B. A systematic review of common conservative therapies for arm lymphoedema secondary to breast cancer treatment. Ann. Oncol. 2007, 18, 639–646.
56. Naik, M.; Nayak, P.; Kumar, K.D. Effect of physiotherapy in the prevention and relief of secondary lymphoedema in subjects with postoperative breast cancer: a systematic review of randomised controlled trials. J. Clin. Diagn. Res. 2021, 15, YE01–YE05.
57. Panchik, D.; Masco, S.; Zinnikas, P.; Hillriegel, B.; Lauder, T.; Suttmann, E.; Chinchilli, V.; McBeth, M.; Hermann, W. Effect of exercise on breast cancer-related lymphedema: what the lymphatic surgeon needs to know. J. Reconstr. Microsurg. 2019, 35, 37–45.
58. Ridner, S.H.; Fu, M.R.; Wanchai, A.; Stewart, B.R.; Armer, J.M.; Cormier, J.N. Self-management of lymphedema: a systematic review of the literature from 2004 to 2011. Nurs. Res. 2012, 61, 291–299
59. Romesberg, M.; Tucker, A.; Kuzminski, K.; Tremback-Ball, A. The effects of resistance exercises on secondary lymphedema due to treatment of breast cancer: a review of current literature. J. Womens Health Phys. Therap. 2017, 41, 91–99.
60. Saraswathi, V.; Latha, S.; Niraimathi, K.; Vidhubala, E. Managing lymphedema, increasing range of motion, and quality of life through yoga therapy among breast cancer survivors. Int. J. Yoga 2021, 14, 3–17
61. Wanchai, A.; Armer, J.M. The effects of yoga on breast-cancer-related lymphedema: a systematic review. J. Health Res. 2020, 34, 409–418.

Supplement 9: Characteristics of the excluded RCTs

Study [ref]	Design	Country	N	Reasons for exclusion
Ammitzbøll 2019 [62]	Randomized controlled trial	Denmark	158	Wrong Population
Andersen 2000 [63]	Randomized controlled trial	Denmark	44	Wrong Intervention
Anderson 2012 [64]	Randomized controlled trial	USA	104	Wrong Population
Balzarini 1993 [65]	Randomized controlled trial	Italy	96	Wrong Intervention
Bertelli 1991 [66]	Randomized controlled trial	Italy	74	Wrong Intervention
Bloomquist 2021 [67].	Randomized controlled trial	Denmark	68	Wrong Population
Box 2002 [68]	Randomized controlled trial	Australia	65	Wrong Population
Bracha 2012 [69]	Randomized controlled trial	Israel	16	Wrong comparator
Buchan 2016 [70]	Randomized controlled trial	Australia	41	Wrong comparator
Buzato 2011 [71]	Non-Randomized controlled trial	Brazil	10	Wrong design
Cantarero-Villanueva 2012 [72]	Randomized controlled trial	Spain	66	Wrong Population
Cantarero-Villanueva 2013 [73]	Randomized controlled trial	Spain	68	Wrong Population
Carati 2003 [74]	Randomized controlled trial	Australia	28	Wrong Intervention
Cormie 2013a [75]	Randomized controlled trial	Australia	17	Wrong comparator
Cormie 2016 [76]	Randomized cross-over design	Australia	25	Wrong design/Comparator
Courneya 2007 [77]	Randomized controlled trial	Canada	242	Wrong Population
Courneya 2009 [78]	Randomized controlled trial	United Kingdom	122	Wrong Population
Courneya 2009a [79]	Randomized controlled trial	Canada	122	Wrong Population
Damstra 2009 [80]	Randomized controlled trial	Austria	36	Wrong Intervention/Comparator
Dayes 2013 [81]	Randomized controlled trial	Canada	103	Wrong Intervention/Comparator
Deacon 2019 [82]	Randomized cross-over controlled trial	Australia	19	Wrong design
Di Blasio 2016 [83]	Randomized controlled trial	Italy	16	Wrong Population
Karadibak 2005 [84]	Randomized controlled trial	Turkey	53	Wrong Intervention/Comparator
Dini 1998 [85]	Randomized controlled trial	Italy	80	Wrong Intervention
Dionne 2018 [86]	Non-Randomized controlled trial	Canada	11	Wrong design

Ergin 2017 [87]	Randomized controlled trial	Turkey	63	Wrong Population
Fisher 2014 [88]	Non-Randomized controlled trial	USA	6	Wrong design
Fong 2014 [89]	Non-Randomized controlled trial	China	23	Wrong design
Gautam 2011 [90]	Non-Randomized controlled trial	India	32	Wrong design
Guerreiro Godoy 2011 [91]	Non-Randomized controlled trial	Brazil	28	Wrong design
Guerreiro Godoy 2010 [92]	Non-Randomized controlled trial	Brazil	21	Wrong design
Haghighat 2010 [93]	Randomized controlled trial	USA	112	Wrong Intervention/Comparator
Hamner 2007 [94]	Non-Randomized controlled trial	USA	135	Wrong design
Harris 2012 [95]	Non-Randomized controlled trial	Canada	---	Wrong design
Hayes 2011 [96]	Randomized controlled trial	Australia	194	Wrong Intervention/Comparator
Hayes 2013 [97]	Randomized controlled trial	Australia	194	Wrong Population
Iyer 2018 [98]	Randomized controlled trial	USA	144	Wrong Population
Johansson 1998 [99]	Randomized controlled trial	Sweden	28	Wrong Intervention/Comparator
Johansson 2005 [100]	Randomized controlled trial	Sweden	31	Wrong comparator
Johansson 2014 [101]	Non-Randomized controlled trial	Sweden	23	Wrong Design
Johansson 1999 [102]	Non-Randomized controlled trial	Sweden	38	Wrong Intervention/Comparator
Jönsson 2009 [103]	Non-Randomized controlled trial	Sweden	42	Wrong Design
Jönsson 2014 [104]	Non-Randomized controlled trial	Sweden	35	Wrong Design
Kasseroller 2010 [105]	Randomized controlled trial	Austria	61	Wrong Intervention/Comparator
Katz 2010 [106]	Non-Randomized controlled trial	USA	10	Wrong Population
Kaviani 2006 [107]	Randomized controlled trial	Iran	11	Wrong Intervention/Comparator
Kilbreath 2012 [108]	Randomized controlled trial	Australia	160	Wrong Population
Kilbreath 2006 [109]	Randomized controlled trial	Australia	22	Wrong Population
King 2012 [110]	Randomized controlled trial	Canada	21	Wrong Intervention/Comparator

Kozanoglu 2009 [111]	Randomized controlled trial	Turkey	47	Wrong Intervention/Comparator
Maher 2012 [112]	Randomized controlled trial	Australia	30	Wrong Intervention/Comparator
Maldonado 2011 [113]	Non-Randomized controlled trial	México	20	Wrong Intervention/Comparator
Malicka 2011 [114]	Randomized controlled trial	Poland	23	Wrong Population
Malicka 2014 [115]	Randomized controlled trial	Poland	28	Wrong Intervention
Mcneely 2004 [116]	Randomized controlled trial	Canada	50	Wrong Intervention/Comparator
Naughton 2021 [117]	Randomized controlled trial	USA	547	Wrong Population
Odynets 2018 [118]	Randomized controlled trial	Ukraine	68	Wrong Population
Odynets 2019c [119]	Randomized controlled trial	Ukraine	115	Wrong Comparator
Odynets 2018d [120]	Randomized controlled trial	Ukraine	115	Wrong Comparator
Ohira 2006 [121]	Randomized controlled trial	USA	86	Wrong Population
Pilch 2009 [122]	Randomized controlled trial	Poland	57	Wrong Intervention/Comparator
Randheer 2011 [123]	Randomized controlled trial	India	25	Wrong Design
Ridner 2012 [124]	Randomized controlled trial	USA	42	Wrong Intervention/Comparator
Ridner 2020 [125]	Randomized controlled trial	USA	46	Wrong Intervention/Comparator
Sagen 2009 [126]	Randomized controlled trial	Norway	204	Wrong Population
Schmitz 2005 [127]	Randomized controlled trial	USA	85	Wrong Population
Schmitz 2009a [128]	Protocol	USA	295	Wrong Design
Simonavice 2017 [129]	Non-Randomized controlled trial	USA	27	Wrong Design
Singh 2016 [130]	Systematic review and Meta-analysis	Australia	---	Wrong Design
Sitzia 2002 [131]	Randomized controlled trial	United Kingdom	28	Wrong Intervention/Comparator
Smoot 2014 [132]	Sub-study of a cross-sectional	USA	133	Wrong Design
Stout 2008 [133]	Case–control study	USA	196	Wrong Design
Szuba 2002 [134]	Randomized controlled trial	USA	23	Wrong Intervention/Comparator
Uzkeser 2013 [135]	Randomized controlled trial	Turkey	31	Wrong Intervention/Comparator
Vale 2011 [136]	Non-Randomized controlled trial	Brazil	18	Wrong Design
Wilburn 2006 [137]	Randomized, crossover study	USA	10	Wrong Design
Williams 2002 [138]	Randomized, crossover study	United Kingdom	31	Wrong Design

Guerreiro Godoy 2012[139]	Randomized, crossover study	Brazil	20	Wrong Design
Lindquist 2015 [140]	Non-Randomized controlled trial	Sweden	109	Wrong Design
Costa Luz 2018 [141]	Non-Randomized controlled trial	Brazil	42	Wrong Design
Ahmed 2006 [142]	Randomized controlled trial	USA	85	Wrong Population
Bok 2016 [143]	Randomized controlled trial	Korea	32	Wrong Outcome
Kilbreath 2020 [144]	Randomized controlled trial	Australia	89	Wrong Population
Schmitz 2019 [145]	Randomized controlled trial	USA	351	Wrong Outcome
Hayes 2009 [146]	Randomized controlled trial	Australia	32	Wrong Outcome
Irdesel 2007 [147]	Randomized controlled trial	Turkey	19	Wrong Comparator
Omar 2020 [148]	Randomized controlled trial	Egypt	60	Wrong Comparator

References:

62. Ammitzbøll, G.; Johansen, C.; Lanng, C.; Andersen, E.W.; Kroman, N.; Zerahn, B.; Hyldegaard, O.; Wittenkamp, M.C.; Dalton, S.O. Progressive resistance training to prevent arm lymphedema in the first year after breast cancer surgery: results of a randomized controlled trial. Cancer 2019, 125, 1683–1692.
63. Andersen, L.; Højris, I.; Erlandsen, M.; Andersen, J. Treatment of breast-cancer-related lymphedema with or without manual lymphatic drainage: a randomized study. Acta Oncol. 2000, 39, 399–405.
64. Anderson, R.T.; Kimmick, G.G.; McCoy, T.P.; Hopkins, J.; Levine, E.; Miller, G.; Ribisl, P.; Mihalko, S.L. A randomized trial of exercise on well-being and function following breast cancer surgery: the RESTORE trial. J. Cancer Surviv. 2012, 6, 172–181.
65. Balzarini, A.; Pirovano, C.; Diazzi, G.; Olivieri, R.; Ferla, F.; Galperti, G.; Sensi, S.; Martino, G. Ultrasound therapy of chronic arm lymphedema after surgical treatment of breast cancer. Lymphology 1993, 26, 128–134.
66. Bertelli, G.; Venturini, M.; Forno, G.; Macchiavello, F.; Dini, D. Conservative treatment of postmastectomy lymphedema: a controlled, randomized trial. Ann. Oncol. 1991, 2, 575–578.
67. Bloomquist, K.; Krustrup, P.; Fristrup, B.; Sørensen, V.; Helge, J.W.; Helge, E.W.; Vadstrup, E.S.; Rørth, M.; Hayes, S.C.; Uth, J. Effects of football fitness training on lymphedema and upper-extremity function in women after treatment for breast cancer: a randomized trial. Acta Oncol. 2021, 60, 392–400.
68. Box, R.C.; Reul-Hirche, H.M.; Bullock-Saxton, J.E.; Furnival, C.M. Physiotherapy after breast cancer surgery: results of a randomised controlled study to minimise lymphoedema. Breast Cancer Res. Treat. 2002, 75, 51–64.
69. Bracha, J.; Katz-Leurer, M. The immediate effect of upper arm exercise compared with lower or combined upper and lower arm exercise on arm volume reduction in women with breast cancer related lymphedema: a randomized preliminary study. Rehabil. Oncol. 2012, 30, 10–15.
70. Buchan, J.; Janda, M.; Box, R.; Schmitz, K.; Hayes, S. A randomized trial on the effect of exercise mode on breast cancer-related lymphedema. Med. Sci. Sports Exerc. 2016,
71. Buzato, E.; Barufi, S.; Dias Guimarães, T.; Amador Franco Brigidio, P. Exercises using leisure resources to reduce arm lymphedema. J. Phlebol. Lymphol. 2011, 4, 17–20.
72. Cantarero-Villanueva, I.; Fernández-Lao, C.; Fernández-de-las-Peñas, C.; López-Barajas, I.B.; Del-Moral-Ávila, R.; de la-Llave-Rincón, A.I.; Arroyo-Morales, M. Effectiveness of water physical therapy on pain, pressure pain sensitivity, and myofascial trigger points in breast cancer survivors: a randomized, controlled clinical trial. Pain Med. 2012, 13, 1509–1519.
73. Cantarero-Villanueva, I.; Fernández-Lao, C.; Cuesta-Vargas, A.I.; Del Moral-Avila, R.; Fernández-de-las-Peñas, C.; Arroyo-Morales, M. The effectiveness of a deep water aquatic exercise program in cancer-related fatigue in breast cancer survivors: a randomized controlled trial. Arch. Phys. Med. Rehabil. 2013, 94, 221–230.
74. Carati, C.J.; Anderson, S.N.; Gannon, B.J.; Piller, N.B. Treatment of postmastectomy lymphedema with low-level laser therapy: a double blind, placebo-controlled trial. Cancer 2003, 98, 1114–1122.
75. Cormie, P.; Galvão, D.A.; Spry, N.; Newton, R.U. Neither heavy nor light load resistance exercise acutely exacerbates lymphedema in breast cancer survivor. Integr. Cancer Ther. 2013, 12, 423–432.
76. Cormie, P.; Singh, B.; Hayes, S.; Peake, J.M.; Galvão, D.A.; Taaffe, D.R.; Spry, N.; Nosaka, K.; Cornish, B.; Schmitz, K.H.; Newton, R.U. Acute inflammatory response to low-, moderate-, and high-load resistance exercise in women with breast cancer-related lymphedema. Integr. Cancer Ther. 2016, 15, 308–317.
77. Courneya, K.S.; Segal, R.J.; Mackey, J.R.; Gelmon, K.; Reid, R.D.; Friedenreich, C.M.; Ladha, A.B.; Proulx, C.; Vallance, J.K.H.; Lane, K.; Yasui, Y.; McKenzie, D.C. Effects of aerobic and resistance exercise in breast cancer patients receiving adjuvant chemotherapy: a multicenter randomized controlled trial. J. Clin. Oncol. 2007, 25, 4396–4404.
78. Courneya, K.S.; Sellar, C.M.; Stevinson, C.; McNeely, M.L.; Peddle, C.J.; Friedenreich, C.M.; Tankel, K.; Basi, S.; Chua, N.; Mazurek, A.; Reiman, T. Randomized controlled trial of the effects of aerobic exercise on physical functioning and

quality of life in lymphoma patients. J. Clin. Oncol. 2009, 27, 4605–4612.
79. Courneya, K.S.; Sellar, C.M.; Stevinson, C.; McNeely, M.L.; Friedenreich, C.M.; Peddle, C.J.; Basi, S.; Chua, N.; Tankel, K.; Mazurek, A.; Reiman, T. Moderator effects in a randomized controlled trial of exercise training in lymphoma patients. Cancer Epidemiol. Biomark. Prev. 2009, 18, 2600–2607.
80. Damstra, R.J.; Partsch, H. Compression therapy in breast cancer-related lymphedema: a randomized, controlled comparative study of relation between volume and interface pressure changes. J. Vasc. Surg. 2009, 49, 1256–1263.
81. Dayes, I.S.; Whelan, T.J.; Julian, J.A.; Parpia, S.; Pritchard, K.I.; D'Souza, D.P.; Kligman, L.; Reise, D.; LeBlanc, L.; McNeely, M.L.; Manchul, L.; Wiernikowski, J.; Levine, M.N. Randomized trial of decongestive lymphatic therapy for the treatment of lymphedema in women with breast cancer. J. Clin. Oncol. 2013, 31, 3758–3763.
82. Deacon, R.; de Noronha, M.; Shanley, L.; Young, K. Does the speed of aquatic therapy exercise alter arm volume in women with breast cancer related lymphoedema? A cross-over randomized controlled trial. Braz. J. Phys. Ther. 2019, 23, 140–147.
83. Di Blasio, A.; Morano, T.; Napolitano, G.; Bucci, I.; Di Santo, S.; Gallina, S.; Cugusi, L.; Di Donato, F.; D'Arielli, A.; Cianchetti, E. Nordic walking and the Isa method for breast cancer survivors: effects on upper limb circumferences and total body extracellular water — a pilot study. Breast Care 2016, 11, 428–431.
84. Karadibak, D.; Yurdalan, S.U.; Saydam, S.; Arican, Z. The comparison of two different physiotherapy methods in treatment of lymphedema after breast surgery. Breast Cancer Res. Treat. 2005, 93, 49–54.
85. Dini, D.; Del Mastro, L.; Gozza, A.; Lionetto, R.; Garrone, O.; Forno, G.; Vidili, G.; Bertelli, G.; Venturini, M. The role of pneumatic compression in the treatment of postmastectomy lymphedema: a randomized phase III study. Ann. Oncol. 1998, 9, 187–190.
86. Dionne, A.; Goulet, S.; Leone, M.; Comtois, A.S. Aquatic exercise training outcomes on functional capacity, quality of life, and lower limb lymphedema: pilot study. J. Altern. Complement. Med. 2018, 24, 1007–1009.
87. Ergin, G.; Karadibak, D.; Sener, H.O.; Gurpinar, B. Effects of aqua-lymphatic therapy on lower extremity lymphedema: a randomized controlled study. Lymphat. Res. Biol. 2017, 15, 284–291.
88. Fisher, M.I.; Donahoe-Fillmore, B.; Leach, L.; O'Malley, C.; Paeplow, C.; Prescott, T.; Merriman, H. Effects of yoga on arm volume among women with breast cancer related lymphedema: a pilot study. J. Bodyw. Mov. Ther. 2014, 18, 559–565.
89. Fong, S.S.M.; Ng, S.S.M.; Luk, W.S.; Chung, J.W.Y.; Ho, J.S.C.; Ying, M.; Ma, A.W.W. Effects of qigong exercise on upper limb lymphedema and blood flow in survivors of breast cancer: a pilot study. Integr. Cancer Ther. 2014, 13, 54–61.
90. Gautam, A.P.; Maiya, A.G.; Vidyasagar, M.S. Effect of home-based exercise program on lymphedema and quality of life in female postmastectomy patients: pre-post intervention study. J. Rehabil. Res. Dev. 2011, 48, 1261–1268.
91. Guerreiro Godoy, M.F.; Guimaraes, T.D.; Oliani, A.H.; Pereira de Godoy, J.M. Association of Godoy & Godoy contention with mechanism with apparatus-assisted exercises in patients with arm lymphedema after breast cancer. Int. J. Gen. Med. 2011, 4, 373–376.
92. Guerreiro Godoy, M.F.; Oliani, A.H.; Pereira de Godoy, J.M. Active exercises utilizing a facilitating device in the treatment of lymphedema resulting from breast cancer therapy. GMS Ger. Med. Sci. 2010, 8, Doc31.
93. Haghighat, S.; Lotfi-Tokaldany, M.; Yunesian, M.; Akbari, M.E.; Nazemi, F.; Weiss, J. Comparing two treatment methods for post mastectomy lymphedema: complex decongestive therapy alone and in combination with intermittent pneumatic compression. Lymphology 2010, 43, 25–33.
94. Hamner, J.B.; Fleming, M.D. Lymphedema therapy reduces the volume of edema and pain in patients with breast cancer. Ann. Surg. Oncol. 2007, 14, 1904–1908.
95. Harris, S.R. "We're all in the same boat": a review of the benefits of dragon boat racing for women living with breast cancer. Evid.-Based Complement. Altern. Med. 2012, 2012, 167651.
96. Hayes, S.C.; Speck, R.M.; Reimet, E.; Stark, A.; Schmitz, K.H. Does the effect of weight lifting on lymphedema following breast cancer differ by diagnostic method: results from a randomized controlled trial. Breast Cancer Res. Treat. 2011, 130, 227–234.
97. Hayes, S.C.; Rye, S.; DiSipio, T.; Yates, P.; Bashford, J.; Pyke, C.; Saunders, C.; Battistutta, D.; Eakin, E. Exercise for health: a randomized, controlled trial evaluating the impact of a pragmatic, translational exercise intervention on the quality of life, function and treatment-related side effects following breast cancer. Breast Cancer Res. Treat. 2013, 137, 175–186.
98. Iyer, N.S.; Cartmel, B.; Friedman, L.; Li, F.; Zhou, Y.; Ercolano, E.; Harrigan, M.; Gottlieb, L.; McCorkle, R.; Schwartz, P.E.; Irwin, M.L. Lymphedema in ovarian cancer survivors: assessing diagnostic methods and the effects of physical activity. Cancer 2018, 124, 1929–1937.
99. Johansson, K.; Lie, E.; Ekdahl, C.; Lindfeldt, J. A randomized study comparing manual lymph drainage with sequential pneumatic compression for treatment of postoperative arm lymphedema. Lymphology 1998, 31, 56–64.
100. Johansson, K.; Tibe, K.; Weibull, A.; Newton, R.U. Low intensity resistance exercise for breast cancer patients with arm lymphedema with or without compression sleeve. Lymphology 2005, 38, 167–180.
101. Johansson, K.; Klernäs, P.; Weibull, A.; Mattsson, S. A home-based weight lifting program for patients with arm lymphedema following breast cancer treatment: a pilot and feasibility study. Lymphology 2014, 47, 51–64.
102. Johansson, K.; Albertsson, M.; Ingvar, C.; Ekdahl, C. Effects of compression bandaging with or without manual lymph drainage treatment in patients with postoperative arm lymphedema. Lymphology 1999, 32, 103–110.
103. Jönsson, C.; Johansson, K. Pole walking for patients with breast cancer-related arm lymphedema. Physiother. Theory Pract. 2009, 25, 165–173.
104. Jönsson, C.; Johansson, K. The effects of pole walking on arm lymphedema and cardiovascular fitness in women treated for breast cancer: a pilot and feasibility study. Physiother. Theory Pract. 2014, 30, 236–242.
105. Kasseroller, R.G.; Brenner, E. A prospective randomised study of alginate-drenched low stretch bandages as an alternative to conventional lymphologic compression bandaging. Support. Care Cancer 2010, 18, 343–350.
106. Katz, E.; Dugan, N.L.; Cohn, J.C.; Chu, C.; Smith, R.G.; Schmitz, K.H. Weight lifting in patients with lower-extremity lymphedema secondary to cancer: a pilot and feasibility study. Arch. Phys. Med. Rehabil. 2010, 91, 1070–1076.
107. Kaviani, A.; Fateh, M.; Yousefi-Nooraie, R.; Alinagi-Zadeh, M.R.; Ataie-Fashtami, L. Low-level laser therapy in management of postmastectomy lymphedema. Lasers Med. Sci. 2006, 21, 90–94.
108. Kilbreath, S.L.; Refshauge, K.M.; Beith, J.M.; Ward, L.C.; Lee, M.; Simpson, J.M.; Hansen, R. Upper limb progressive resistance training and stretching exercises following surgery for early breast cancer: a randomized controlled trial. Breast Cancer Res. Treat. 2012, 133, 667–676.
109. Kilbreath, S.; Refshauge, K.; Beith, J.; Lee, M. Resistance and stretching shoulder exercises early following axillary surgery for breast cancer. Rehabil. Oncol. 2006, 24(2), 9–14.
110. King, M.; Deveaux, A.; White, H.; Rayson, D. Compression garments versus compression bandaging in decongestive lymphatic therapy for breast cancer-related lymphedema: a randomized controlled trial. Support. Care Cancer 2012, 20,

1031–1036.
111. Kozanoglu, E.; Basaran, S.; Paydas, S.; Sarpel, T. Efficacy of pneumatic compression and low-level laser therapy in the treatment of postmastectomy lymphoedema: a randomized controlled trial. Clin. Rehabil. 2009, 23, 117–124.
112. Maher, J.; Refshauge, K.; Ward, L.; Paterson, R.; Kilbreath, S. Change in extracellular fluid and arm volumes as a consequence of a single session of lymphatic massage followed by rest with or without compression. Support. Care Cancer 2012, 20, 3079–3086.
113. Maldonado, G.E.M.; Pérez, C.A.A.; Covarrubias, E.E.A.; Cabriales, S.A.M.; Leyva, L.A.; Pérez, J.C.J.; de la Garza-Salazar, J.G. Autologous stem cells for the treatment of post-mastectomy lymphedema: a pilot study. Cytotherapy 2011, 13, 1249–1255.
114. Malicka, I.; Stefańska, M.; Rudziak, M.; Jarmoluk, P.; Pawłowska, K.; Szczepańska-Gieracha, J.; Woźniewski, M. The influence of Nordic walking exercise on upper extremity strength and the volume of lymphoedema in women following breast cancer treatment. Isokinet. Exerc. Sci. 2011, 19, 295–304.
115. Malicka, I.; Rosseger, A.; Hanuszkiewicz, J.; Woźniewski, M. Kinesiology taping reduces lymphedema of the upper extremity in women after breast cancer treatment: a pilot study. Prz. Menopauzalny 2014, 13, 221–226.
116. McNeely, M.L.; Magee, D.J.; Lees, A.W.; Bagnall, K.M.; Haykowsky, M.; Hanson, J. The addition of manual lymph drainage to compression therapy for breast cancer related lymphedema: a randomized controlled trial. Breast Cancer Res. Treat. 2004, 86, 95–106.
117. Naughton, M.J.; Liu, H.; Seisler, D.K.; Le-Rademacher, J.; Armer, J.M.; Oliveri, J.M.; Sloan, J.A.; Hock, K.; Schwartz, M.; Unzeitig, G.; Melnik, M.; Yee, L.D.; Fleming, G.F.; Taylor, J.R.; Loprinzi, C.; Paskett, E.D. Health-related quality of life outcomes for the LEAP study — CALGB 70305 (Alliance): a lymphedema prevention intervention trial for newly diagnosed breast cancer patients. Cancer 2021, 127, 300–309.
118. Odynets, T.; Briskin, Y.; Perederiy, A.; Pityn, M.; Svistelnyk, I. Effect of water physical therapy on quality of life in breast cancer survivors. Physiother. Q. 2018, 26(4), 11–16.
119. Odynets, T.; Briskin, Y.; Todorova, V. Effects of different exercise interventions on quality of life in breast cancer patients: a randomized controlled trial. Integr. Cancer Ther. 2019, 18, 1534735419880598.
120. Odynets, T.; Briskin, Y.; Sydorko, O.; Tyshchenko, V.; Putrov, S. Effectiveness of individualized physical rehabilitation programs on post-mastectomy pain in breast cancer survivors. Physiother. Q. 2018, 26(3), 1–5.
121. Ohira, T.; Schmitz, K.H.; Ahmed, R.L.; Yee, D. Effects of weight training on quality of life in recent breast cancer survivors: the Weight Training for Breast Cancer Survivors (WTBS) study. Cancer 2006, 106, 2076–2083.
122. Pilch, U.; Woźniewski, M.; Szuba, A. Influence of compression cycle time and number of sleeve chambers on upper extremity lymphedema volume reduction during intermittent pneumatic compression. Lymphology 2009, 42, 26–35.
123. Randheer, S.; Kadambari, D.; Srinivasan, K.; Bhuvaneswari, V.; Bhanumathy, M.; Salaja, R. Comprehensive decongestive therapy in postmastectomy lymphedema: an Indian perspective. Indian J. Cancer 2011, 48, 397–402.
124. Ridner, S.H.; Murphy, B.; Deng, J.; Kidd, N.; Galford, E.; Bonner, C.; Bond, S.M.; Dietrich, M.S. A randomized clinical trial comparing advanced pneumatic truncal, chest, and arm treatment to arm treatment only in self-care of arm lymphedema. Breast Cancer Res. Treat. 2012, 131, 147–158.
125. Ridner, S.H.; Dietrich, M.S.; Davis, A.J.; Sinclair, V. A randomized clinical trial comparing the impact of a web-based multimedia intervention versus an educational pamphlet on patient outcomes in breast cancer survivors with chronic secondary lymphedema. J. Womens Health 2020, 29, 734–744.
126. Sagen, Å.; Kåresen, R.; Risberg, M.A. Physical activity for the affected limb and arm lymphedema after breast cancer surgery: a prospective, randomized controlled trial with two years follow-up. Acta Oncol. 2009, 48, 1102–1110.
127. Schmitz, K.H.; Ahmed, R.L.; Hannan, P.J.; Yee, D. Safety and efficacy of weight training in recent breast cancer survivors to alter body composition, insulin, and insulin-like growth factor axis proteins. Cancer Epidemiol. Biomark. Prev. 2005, 14, 1672–1680.
128. Schmitz, K.H.; Troxel, A.B.; Cheville, A.; Grant, L.L.; Bryan, C.J.; Gross, C.R.; Lytle, L.A.; Ahmed, R.L. Physical activity and lymphedema (the PAL trial): assessing the safety of progressive strength training in breast cancer survivors. Contemp. Clin. Trials 2009, 30, 233–245.
129. Simonavice, E.; Kim, J.S.; Panton, L. Effects of resistance exercise in women with or at risk for breast cancer-related lymphedema. Support. Care Cancer 2017, 25, 9–15.
130. Singh, B.; Buchan, J.; Box, R.; Janda, M.; Peake, J.; Purcell, A.; Reul-Hirche, H.; Hayes, S.C. Compression use during an exercise intervention and associated changes in breast cancer-related lymphedema. Asia Pac. J. Clin. Oncol. 2016, 12, 216–224.
131. Sitzia, J.; Sobrido, L.; Harlow, W. Manual lymphatic drainage compared with simple lymphatic drainage in the treatment of post-mastectomy lymphoedema: a pilot randomised trial. Physiotherapy 2002, 88, 99–107.
132. Smoot, B.; Zerzan, S.; Krasnoff, J.; Wong, J.; Cho, M.; Dodd, M. Upper extremity bioimpedance before and after treadmill testing in women post breast cancer treatment. Breast Cancer Res. Treat. 2014, 148, 445–453.
133. Stout Gergich, N.L.; Pfalzer, L.A.; McGarvey, C.; Springer, B.; Gerber, L.H.; Soballe, P. Preoperative assessment enables the early diagnosis and successful treatment of lymphedema. Cancer 2008, 112, 2809–2819.
134. Szuba, A.; Achalu, R.; Rockson, S.G. Decongestive lymphatic therapy for patients with breast carcinoma-associated lymphedema: a randomized, prospective study of a role for adjunctive intermittent pneumatic compression. Cancer 2002, 95, 2260–2267.
135. Uzkeser, H.; Karatay, S. Intermittent pneumatic compression pump in upper extremity impairments of breast cancer-related lymphedema. Turk. J. Med. Sci. 2013, 43, 99–103.
136. Vale, T.C.P.; Guimarães, T.D.; Libanori, D.; Baruffi, S.M. Synergistic effect of low elastic compression sleeves in the treatment of lymphedema after breast cancer treatment. J. Phlebol. Lymphol. 2011, 4(1), 5–9.
137. Wilburn, O.; Wilburn, P.; Rockson, S.G. A pilot, prospective evaluation of a novel alternative for maintenance therapy of breast cancer-associated lymphedema. BMC Cancer 2006, 6, 84.
138. Williams, A.F.; Vadgama, A.; Franks, P.J.; Mortimer, P.S. A randomized controlled crossover study of manual lymphatic drainage therapy in women with breast cancer-related lymphoedema. Eur. J. Cancer Care 2002, 11, 254–261.
139. Guerreiro Godoy, M.F.; Pereira, M.R.; Oliani, A.H.; de Godoy, J.M.P. Synergic effect of compression therapy and controlled active exercises using a facilitating device in the treatment of arm lymphedema. Int. J. Med. Sci. 2012, 9, 280–284.
140. Lindquist, H.; Enblom, A.; Dunberger, G.; Nyberg, T.; Bergmark, K. Water exercise compared to land exercise or standard care in female cancer survivors with secondary lymphedema. Lymphology 2015, 48, 64–79.
141. Luz, R.P.C.; Haddad, C.A.S.; Rizzi, S.K.L.A.; Elias, S.; Nazario, A.C.P.; Facina, G. Complex therapy physical alone or associated with strengthening exercises in patients with lymphedema after breast cancer treatment: a controlled clinical trial. Asian Pac. J. Cancer Prev. 2018, 19, 1405–1410.

142. Ahmed, R.L.; Thomas, W.; Yee, D.; Schmitz, K.H. Randomized controlled trial of weight training and lymphedema in breast cancer survivors. J. Clin. Oncol. 2006, 24, 2765–2772.
143. Bok, S.K.; Jeon, Y.; Hwang, P.S. Ultrasonographic evaluation of the effects of progressive resistive exercise in breast cancer-related lymphedema. Lymphat. Res. Biol. 2016, 14, 18–24.
144. Kilbreath, S.L.; Ward, L.C.; Davis, G.M.; Degnim, A.C.; Hackett, D.A.; Skinner, T.L.; Black, D. Reduction of breast lymphoedema secondary to breast cancer: a randomised controlled exercise trial. Breast Cancer Res. Treat. 2020, 184, 459–467.
145. Schmitz, K.H.; Troxel, A.B.; Dean, L.T.; DeMichele, A.; Brown, J.C.; Sturgeon, K.; Zhang, Z.; Evangelisti, M.; Spinelli, B.; Kallan, M.J.; Denlinger, C.; Cheville, A.; Winkels, R.M.; Chodosh, L.; Sarwer, D.B. Effect of home-based exercise and weight loss programs on breast cancer-related lymphedema outcomes among overweight breast cancer survivors: the WISER Survivor randomized clinical trial. JAMA Oncol. 2019, 5, 1605–1613.
146. Hayes, S.C.; Reul-Hirche, H.; Turner, J. Exercise and secondary lymphedema: safety, potential benefits, and research issues. Med. Sci. Sports Exerc. 2009, 41, 483–489.
147. Irdesel, J.; Kahraman Celiktas, S. Effectiveness of exercise and compression garments in the treatment of breast cancer related lymphedema. Turk. J. Phys. Med. Rehabil. 2007, 53, 16–21.
148. Omar, M.T.A.; Gwada, R.F.M.; Omar, G.S.M.; EL-Sabagh, R.M.; Mersal, A.E.A.E. Low-intensity resistance training and compression garment in the management of breast cancer-related lymphedema: single-blinded randomized controlled trial. J. Cancer Educ. 2020, 35, 1101–1110.

Supplement S10: Additional information for the characteristics of the systematic reviews and metanalyses included.

Autor/año	Country	Outcome	Funding	Registration info	Conclusion
Karki 2009	Finland	-Lymphedem	No conflicts of interest regarding the publication of this paper	Not registered	Compression bandages can reduce lymphedema in patients with breast cancer, but better evidence is required for other therapies.
Lian 2024	Ireland	-Lymphedema severity	No conflicts of interest regarding the publication of this paper	Not registered	High frequency exercise, like resistance and yoga, can reduce the severity of the lymphedema. Training at home is effective, but continual practice is required to maintain benefits.
Muñoz-Gómez 2023	Spain	-Fatigue<br>-Pain<br>-Lymphedema<br>-Quality of life.	No conflicts of interest regarding the publication of this paper	Not registered	Therapeutic aquatic exercise improves fatigue, pain, and quality of life in breast cancer survivors, with high adherence and without adverse effects, although its impact in lymphedema continues to be unclear.
Rogan 2016	Switzerland	-Reduction of volume or oedema	No conflicts of interest regarding the publication of this paper	PROSPERO (CRD42014010700)	Exercise and intermittent pneumatic compression can reduce oedema in BCRL, while compression sleeves prevent additional increase but do not reduce volume in the acute phase.
Paramanandam 2014	United Kingdom	-lymphoedema onset or exacerbation<br>-Limb strength<br>-Quality of life<br>-Body mass index	No conflicts of interest regarding the publication of this paper	PROSPERO (CRD42012002737)	Weight training is safe and improves strength and quality of life in women with lymphedema or at risk. Factors such as compression, supervision, and intensity can influence, but more evidence is needed.
Singh 2016	Australia	-Lymphedema<br>-Symptoms associated with lymphedema (pain, heaviness, tightness in the affected extremity)	Unclear conflict of interest	Not registered	Progressive regular exercise is safe for people with secondary lymphedema, without worsening their symptoms. However, the evidence on the use of compression garments during exercise is insufficient.
Hayes 2022	Australia	-Lymphedema<br>-Symptoms associated with CRL<br>-Symptoms associated with lymphedema	No conflicts of interest regarding the publication of this paper	PROSPERO (CRD42020196623)	Aerobic and resistance exercise is recommendable for people with or at risk of CRL, including without supervision, according to the response in the symptoms.
Wanchai 2018	Thailand	-Lymphedema	No conflicts of interest regarding the publication of this paper	Not registered	Supervise resistance exercise is safe and beneficial for people with or at risk of BCRL, but more research is necessary to confirm these findings.
Naghibi 2018	Iran	-Clinical effectiveness	Unclear conflict of interest	No registered	Lymphedema continues to be a problem for breast cancer survivors. Exercise does not seem to affect arm volume, but does improve quality of life.

Supplement S11: Overall results of CCA

Overall results		
Number of columns	c	9
Number of rows	r	105
Number of included primary studies	N	170
Covered area	N/(rc)	17,99%
Corrected covered area	(N-r)/(rc-r)	7.74
Interpretation of overlap	Moderate overlap	
Corrected covered area.<br>(Adjusting by structural zeros)	(N-r)/(rc-r-X)	7,74%

Supplement S12: Evaluation of risk of bias in the RCTs

a) Volumetric changes in arm:

-Lymphedema volume < 6 months:

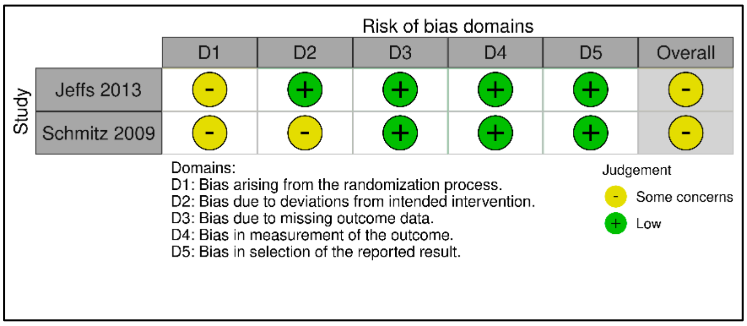

-Volume reduction < 6 months:

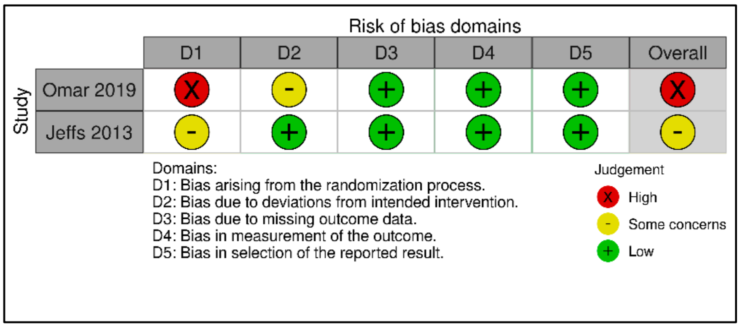

:

-Per cent reduction < 6 months:

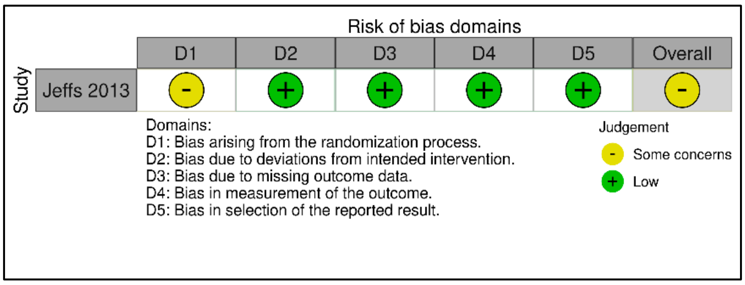

b) Global quality of life < 6 months:

Risk of bias domains

Study	D1	D2	D3	D4	D5	Overall
Do 2015	-	+	+	+	+	-
Jeffs 2013	-	+	+	+	+	-
Loundon 2013	X	+	X	+	+	X
Pasyar 2019	-	X	+	+	+	X

Domains:
D1: Bias arising from the randomization process.
D2: Bias due to deviations from intended intervention.
D3: Bias due to missing outcome data.
D4: Bias in measurement of the outcome.
D5: Bias in selection of the reported result.

Judgement
X High
- Some concerns
+ Low

Subscales of Quality of life:

-Physical functioning < 6 months:

Risk of bias domains

Study	D1	D2	D3	D4	D5	Overall
Do 2015	-	+	+	+	+	-
Jeffs 2013	-	+	+	+	+	-
Loundon 2013	X	+	X	+	+	X
Pasyar 2019	-	X	+	+	+	X
Cormie 2013	-	+	+	+	+	-
Kim 2010	-	+	+	-	+	-
Mckenzie 2003	-	-	+	+	+	-
Tidhar 2010	-	+	+	X	+	X

Domains:
D1: Bias arising from the randomization process.
D2: Bias due to deviations from intended intervention.
D3: Bias due to missing outcome data.
D4: Bias in measurement of the outcome.
D5: Bias in selection of the reported result.

Judgement
X High
- Some concerns
+ Low

-Role functioning < 6 months:

Risk of bias domains

Study	D1	D2	D3	D4	D5	Overall
Do 2015	-	+	+	+	+	-
Loundon 2013	X	+	X	+	+	X
Pasyar 2019	-	X	+	+	+	X

Domains:
D1: Bias arising from the randomization process.
D2: Bias due to deviations from intended intervention.
D3: Bias due to missing outcome data.
D4: Bias in measurement of the outcome.
D5: Bias in selection of the reported result.

Judgement
X High
- Some concerns
+ Low

-Emotional functioning < 6 months:

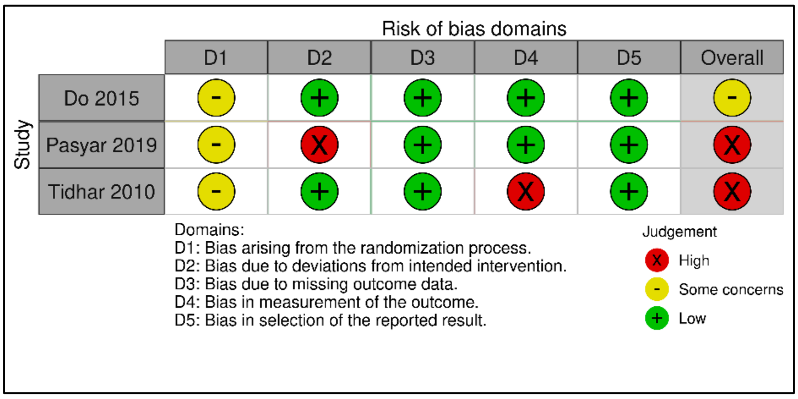

-Social functioning < 6 months:

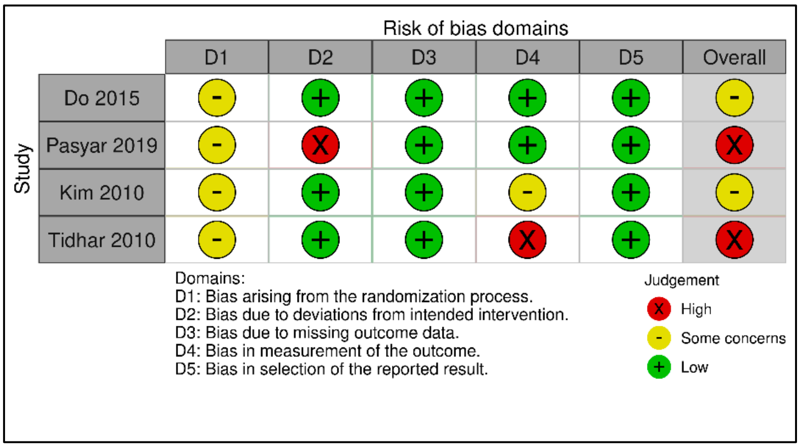

-Mental health < 6 months:

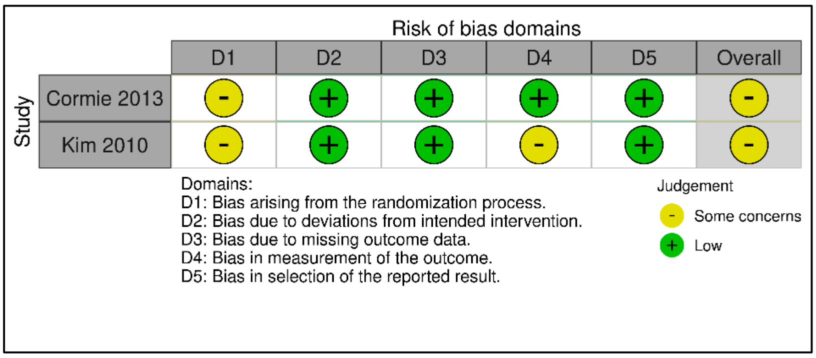

-Mental health > 6 months:

Risk of bias domains

Study	D1	D2	D3	D4	D5	Overall
Speck 2010	-	+	X	X	+	X

Domains:
D1: Bias arising from the randomization process.
D2: Bias due to deviations from intended intervention.
D3: Bias due to missing outcome data.
D4: Bias in measurement of the outcome.
D5: Bias in selection of the reported result.

Judgement
X High
- Some concerns
+ Low

c) Pain < 6 months

Risk of bias domains

Study	D1	D2	D3	D4	D5	Overall
Loudon 2013	X	+	X	+	+	X
Letellier 2014	X	X	+	X	+	X

Domains:
D1: Bias arising from the randomization process.
D2: Bias due to deviations from intended intervention.
D3: Bias due to missing outcome data.
D4: Bias in measurement of the outcome.
D5: Bias in selection of the reported result.

Judgement
X High
+ Low

d) Grip strength < 6 months:

Risk of bias domains

Study	D1	D2	D3	D4	D5	Overall
Cormie 2013	-	+	+	+	+	-
Letellier 2014	X	X	+	X	+	X

Domains:
D1: Bias arising from the randomization process.
D2: Bias due to deviations from intended intervention.
D3: Bias due to missing outcome data.
D4: Bias in measurement of the outcome.
D5: Bias in selection of the reported result.

Judgement
X High
- Some concerns
+ Low

e) Range of motion (ROM):

-Wrist flexion < 6 months:

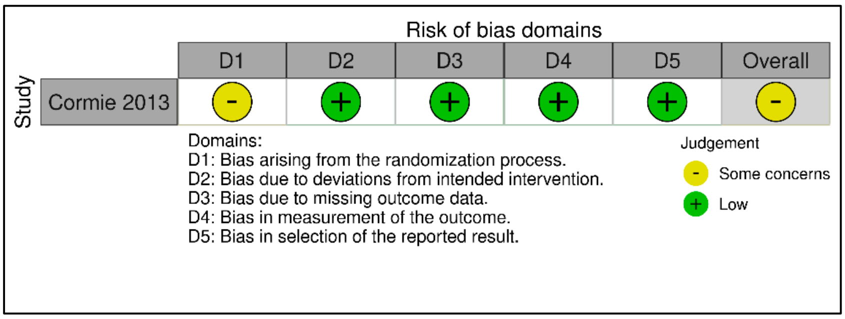

-Wrist extension < 6 months:

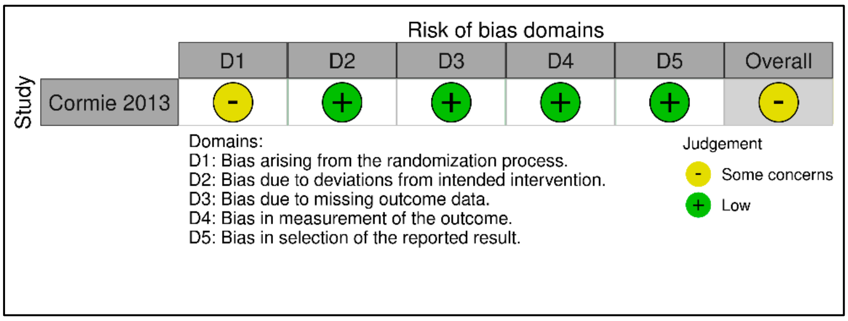

-Elbow flexion < 6 months:

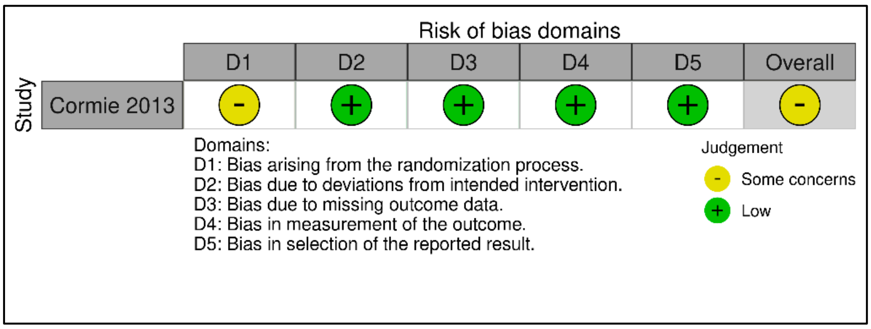

-Elbow extension < 6 months:

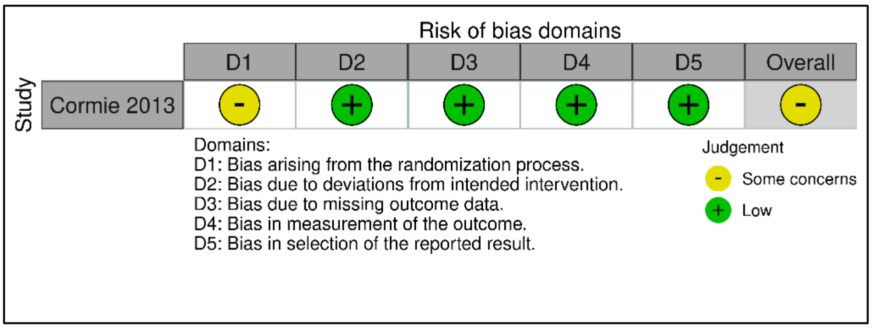

-Shoulder flexion < 6 months:

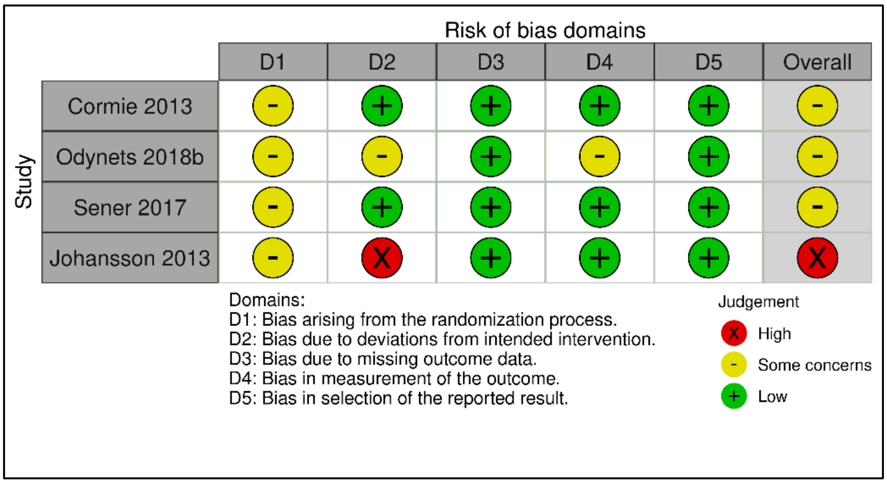

-Shoulder extension < 6 months:

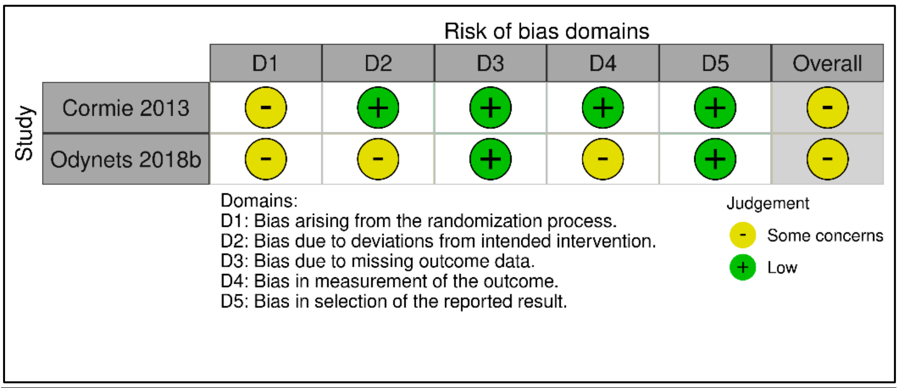

-Shoulder abduction < 6 months:

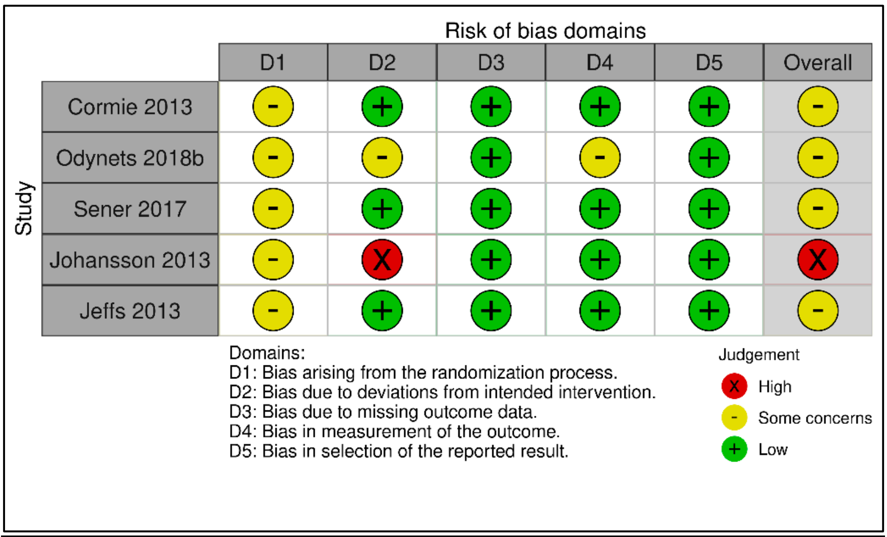

-Internal shoulder rotation < 6 months:

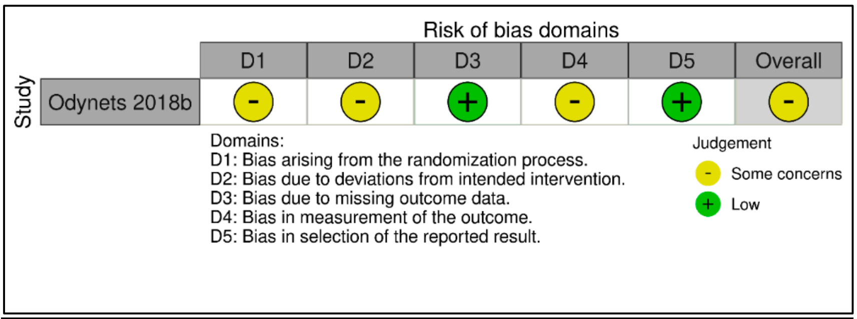

-External shoulder rotation < 6 months:

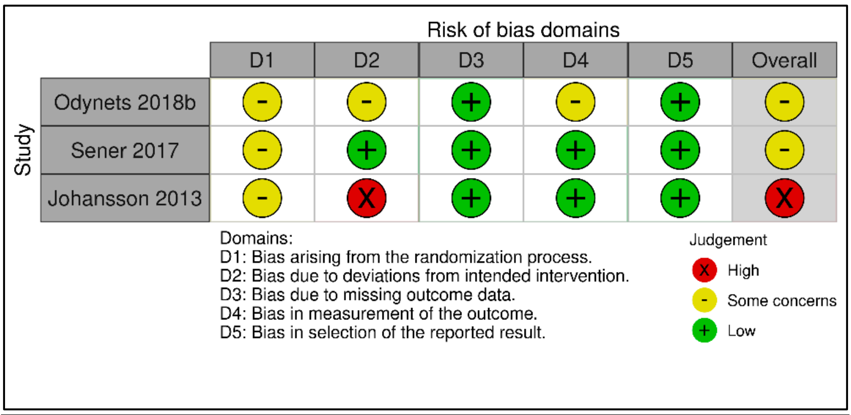

f) <u>Upper limb function < 6 months:</u>

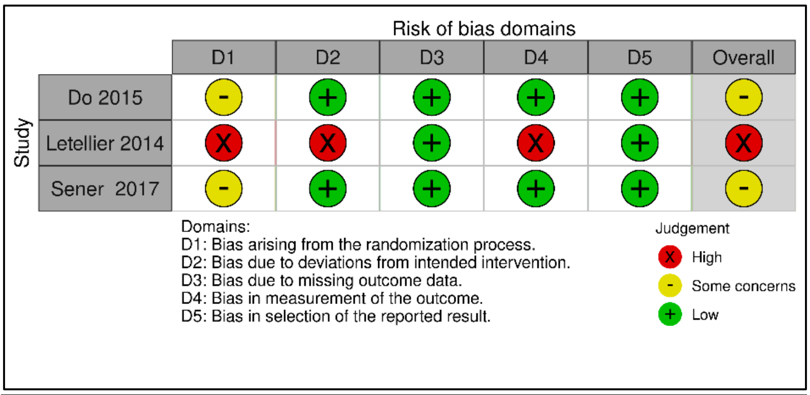

Supplement S13: Summary of effect and certainty of evidence estimators for primary results

**a) Volumetric changes in arm**					
**Outcome: Volume of lymphedema < 6 months**					
**Comparison**	**No. Of studies**	**No. Of participants**	**Effect size (MD IC 95%)**	**GRADE**	**Justification**
Home-based exercise program plus standard lymphoedema self-care vs Usual Care	1	23	-95.24 (-176.03, 14.45)	Very low	Very low certainty due to risk of bias (randomization) and very serious imprecision (wide confidence interval crossing multiple clinical thresholds and small sample).
**Volume of lymphedema > 6 months (≥5% increment)**					
**Comparison**	**No. Of studies**	**No. Of participants**	**Effect size** (RR IC 95%)	**GRADE**	**Justification**
Weight-Lifting Program vs Usual Care	1	139	0.13 (0.07, 0.25)	Moderate	Moderate certainty due to risk of bias. No concerns about inconsistency, indirectness, publication bias, or imprecision.
**Lymphedema Volume > 6 months (≥5% reduction)**					
Weight-Lifting Program vs Usual Care	1	139	0.85 (0.44, 1.66)	Very low	Very low certainty due to risk of bias (randomization and protocol deviations) and very serious imprecision (wide CI crossing thresholds and small sample).
**Outcome: Volume Reduction < 6 months**					
**Comparison**	**No. Of studies**	**No. Of participants**	**Effect size (MD IC 95%)**	**GRADE**	**Justification**
Home-based exercise program plus standard lymphoedema self-care vs Usual Care	1	23	2.08 (-76.06, 80.22)	Very low	Very low certainty due to risk of bias (randomization) and very serious imprecision (wide CI and small sample).
**Outcome: Per cent Reduction < 6 months**					
Home-based exercise program plus standard lymphoedema self-care vs Usual Care	1	23	-3.56 (-15.85, 8.73)	Very low	Very low certainty due to risk of bias (randomization) and very serious imprecision (wide CI and small sample).
**b) Outcome: Global quality of life < 6 months**					
Resistive exercise + CDT vs CDT	1	44	-0.70 (-12.95, 11.55)	Very low	Very low certainty due to risk of bias (randomization concerns) and very serious imprecision (wide CI and small sample size).
Yoga vs Usual care	2	46	0.38 (-2.30, 0.97)	Very low	Very low certainty due to very serious risk of bias and serious imprecision (CI crosses thresholds and small sample).
Home-based exercise program plus standard lymphedema self-care vs Usual Care	1	23	Improvements in QoL not observed		Very low certainty due to risk of bias (randomization concerns) and very serious imprecision (no effect estimate or CI, and small sample).
**Outcome: Quality of life - Physical functioning < 6 months**					
High-load resistance exercise vs Usual care	1	41	3.10 (-2.26, 8.46)	Very low	Very low certainty due to risk of bias (randomization) and very serious imprecision (wide confidence interval crossing multiple thresholds and small sample).
Low-load resistance exercise vs Usual care	1	40	3.80 (-1.10, 8.70)	Very low	Very low certainty due to risk of bias (randomization) and very serious imprecision (wide confidence interval crossing multiple thresholds and small sample).
High-load resistance exercise vs Low-load resistance exercise	1	43	-0.70 (-4.82, 3.42)	Very low	Very low certainty due to risk of bias (randomization) and very serious imprecision (wide CI crossing multiple thresholds and small sample).
Resistance exercise + CDT vs CDT	1	44	8.10 (-3.29, 19.49)	Very low	Very low certainty due to risk of bias (randomization) and very serious imprecision (wide confidence interval crossing multiple clinical thresholds and small sample).
Active resistance exercise (ARE) + CDT vs CDT	1	40	9.12 (0.86, 17.38)	Very low	Very low certainty due to risk of bias (some concerns in randomization and outcome measurement) and very serious imprecision (wide confidence interval crossing effect size thresholds and small sample).
Yoga vs Usual care	1	27	13.00 (6.65, 19.35)	Very low	Very low certainty due to very serious risk of bias (high risk due to protocol deviations and concerns in randomization) and serious imprecision (wide CI and small sample).
Self-care + ALT vs Self care	1	62	-5.20 (-14-18, 3-78)	Very low	Very low certainty due to very serious risk of bias (high risk in outcome measurement and concerns in randomization) and serious imprecision (wide CI crossing clinical thresholds).
Aerobic exercise + resistance vs no intervention	1	14	Without significant improvement		Very low certainty due to risk of bias (randomization and protocol deviations) and very serious imprecision (no effect estimate or confidence interval, and likely small sample).
**Outcome: Quality of life - Role functioning < 6 months**					
Resistance exercise + CDT vs CDT	1	44	-3.40 (-18.85, 12.05)	Very low	Very low certainty due to risk of bias (randomization concerns) and very serious imprecision (extremely wide confidence interval crossing clinical thresholds and small sample).
Yoga vs Usual care	2	46	0.35	Very low	Very low certainty due to very serious risk of bias (high risk from

			(-0.23, 0.94)		protocol deviations and concerns in randomization) and serious imprecision (confidence interval crosses a threshold and sample size is small).
**Outcome: Quality of life - Emotional functioning < 6 months**					
Resistance exercise + CDT vs CDT	1	44	-10.70 (-25.34, 3.94)	Very low	Very low certainty due to risk of bias (randomization concerns) and very serious imprecision (wide confidence interval crossing clinical thresholds and small sample size).
Yoga vs Usual care	1	27	30.28 (9.44, 51.12)	Very low	Very low certainty due to very serious risk of bias (high risk from protocol deviations and concerns in randomization) and serious imprecision (wide confidence interval crossing effect size thresholds and small sample).
Self-care + ALT vs Self care	1	48	-4.00 (-8.43, 0.43)	Very low	Very low certainty due to very serious risk of bias (high risk in outcome measurement and concerns in randomization) and serious imprecision (confidence interval crosses thresholds and sample is small).
**Outcome: Quality of life - Social functioning < 6 months**					
Resistance exercise + CDT vs CDT	1	44	-11.80 (-27.9, 4.30)	Very low	Very low certainty due to risk of bias (randomization concerns) and very serious imprecision (extremely wide confidence interval crossing multiple clinical thresholds and small sample size).
Active Resistance Exercise (ARE) + CDT vs CDT	1	40	3.68 (-2.51, 9.87)	Very low	Very low certainty due to risk of bias (some concerns in randomization and outcome measurement) and very serious imprecision (wide confidence interval crossing clinical thresholds and small sample size).
Yoga vs Usual care	1	27	6.39 (-3.94, 16.72)	Very low	Very low certainty due to very serious risk of bias (high risk from protocol deviations and concerns in randomization) and serious imprecision (confidence interval crosses thresholds and small sample size).
Self-care + ALT vs Self care	1	48	-2.60 (-5.81, 0.61)	Very low	Very low certainty due to very serious risk of bias (high risk in outcome measurement and concerns in randomization) and serious imprecision (wide CI crossing thresholds and small sample size).
**Outcome: Quality of life - Mental health < 6 months**					
High-load resistance exercise vs Usual care	1	41	-0.60 (-7.88, 6.68)	Very low	Very low certainty due to risk of bias (randomization concerns) and very serious imprecision (wide confidence interval crossing clinical thresholds and small sample size).
Low-load resistance exercise vs Usual care	1	40	6.00 (-0.43, 12.43)	Very low	Very low certainty due to risk of bias (randomization concerns) and very serious imprecision (wide confidence interval crossing clinical thresholds and small sample size).
High-load resistance exercise vs Low-load resistance exercise	1	43	-6.60 (-12.40, -0.80)	Very low	Very low certainty due to risk of bias (randomization concerns) and very serious imprecision (confidence interval crossing multiple clinically important thresholds and small sample size).
Active resistive exercise (ARE) + CDT vs CDT	1	40	5.75 (-4.32, 15.82)	Very low	Very low certainty due to risk of bias (some concerns in randomization and outcome measurement) and very serious imprecision (confidence interval crossing multiple clinically important thresholds and small sample size).
**Outcome: Quality of life - Mental health > 6 months**					
Weight training intervention vs No intervention	1	112	1.00 (-2.44, 4.44)	Very low	Very low certainty due to very serious risk of bias (high risk from missing data and outcome measurement, and concerns in randomization) and serious imprecision (confidence interval crossing clinical thresholds).
**Outcome: Pain < 6 months**					
Yoga vs Usual care	1	19	0.24 (-1.25, 1.73)	Very low	Very low certainty due to very serious risk of bias (high risk in randomization and missing data) and very serious imprecision (extremely wide confidence interval crossing clinical thresholds and very small sample).
Aqua lymphatic therapy vs Home Land-based exercise program alone	1	18	Pain reduction in the ALT group	Very low	Very low certainty due to very serious risk of bias (high risk in randomization, missing data, and outcome measurement) and very serious imprecision (no effect estimate or confidence interval and very small sample).

Supplement S14: Summary of effect and certainty of evidence estimators for secondary results

Outcome: Adverse events					
**Comparison**	**No. Of studies**	**No. Of participants**	**Effect size (MD IC 95%)**	**GRADE**	**Justification**
NR					
**Outcome: Grip strength < 6 months**					
High-load resistance exercise vs Usual care	1	41	0.90 (-3.27, 5.07)	Very low	Very low certainty due to risk of bias (randomization concerns) and very serious imprecision (confidence interval crossing multiple clinically important thresholds and small sample size).
Low-load resistance exercise vs Usual care	1	40	0.30 (-3.48, 4.08)	Very low	Very low certainty due to risk of bias (randomization concerns) and very serious imprecision (confidence interval crossing multiple clinically important thresholds and small sample size).
High-load resistance exercise vs Low-load resistance exercise	1	43	0.60 (-3.35, 4.55)	Very low	Very low certainty due to risk of bias (randomization concerns) and very serious imprecision (confidence interval crossing multiple clinically important thresholds and small sample size).
ALT VS Home exercise	1	18	-0.10 (-6.61, 6.41)	Very low	Very low certainty due to very serious risk of bias (high risk in randomization, protocol deviations, and outcome measurement) and very serious imprecision (extremely wide confidence interval and very small sample).
**Outcome: ROM – Wrist flexion < 6 months**					
High-load resistance exercise vs Usual care	1	41	4.60 (-0.64, 9.84)	Very low	Very low certainty due to risk of bias (randomization concerns) and very serious imprecision (confidence interval crossing multiple clinically important thresholds and small sample size).
Low-load resistance exercise vs Usual care	1	40	4.10 (-0.40, 8.60)	Very low	Very low certainty due to risk of bias (randomization concerns) and very serious imprecision (confidence interval crossing multiple clinically important thresholds and small sample size).
High-load resistance exercise vs Low-load resistance exercise	1	43	0.50 (-4.50, 5.50)	Very low	Very low certainty due to risk of bias (randomization concerns) and very serious imprecision (confidence interval crossing multiple clinically important thresholds and small sample size).
**Outcome: ROM – Wrist extension < 6 months**					
High-load resistance exercise vs Usual care	1	41	-0.80 (-6.52, 4.92)	Very low	Very low certainty due to risk of bias (randomization concerns) and very serious imprecision (confidence interval crossing multiple clinically important thresholds and small sample size).
Low-load resistance exercise vs Usual care	1	40	3.50 (-1.93, 8.93)	Very low	Very low certainty due to risk of bias (randomization concerns) and very serious imprecision (confidence interval crossing multiple clinically important thresholds and small sample size).
High-load resistance exercise vs Low-load resistance exercise	1	43	-4.30 (-9.28, 0.68)	Very low	Very low certainty due to risk of bias (randomization concerns) and very serious imprecision (confidence interval crossing multiple clinically important thresholds and small sample size).
**Outcome: ROM – Elbow flexion < 6 months**					
High-load resistance exercise vs Usual care	1	41	-0.60 (-4.17, 2.97)	Very low	Very low certainty due to risk of bias (randomization concerns) and very serious imprecision (confidence interval crossing multiple clinically important thresholds and small sample size).
Low-load resistance exercise vs Usual care	1	40	-2.40 (-6.00, 1.20)	Very low	Very low certainty due to risk of bias (randomization concerns) and very serious imprecision (confidence interval crossing multiple clinically important thresholds and small sample size).
High-load resistance exercise vs Low-load resistance exercise	1	43	1.80 (-1.97, 5.57)	Very low	Very low certainty due to risk of bias (randomization concerns) and very serious imprecision (confidence interval crossing multiple clinically important thresholds and small sample size).
**Outcome: ROM – Elbow extension < 6 months**					
High-load resistance exercise vs Usual care	1	41	-2.00 (-4.63, 0.63)	Very low	Very low certainty due to risk of bias (randomization concerns) and very serious imprecision (confidence interval crossing multiple clinically important thresholds and small sample size).
Low-load resistance exercise vs Usual care	1	40	-0.10 (-2.61, 2.41)	Very low	Very low certainty due to risk of bias (randomization concerns) and very serious imprecision (confidence interval crossing multiple clinically important thresholds and small sample size).
High-load resistance exercise vs Low-load resistance exercise	1	43	-1.90 (-4.66, 0.86)	Very low	Very low certainty due to risk of bias (randomization concerns) and very serious imprecision (confidence interval crossing multiple clinically important thresholds and small sample size).
**Outcome: ROM – Shoulder flexion < 6 months**					
High-load resistance exercise vs Usual care	1	41	-1.40 (-11.83, 9.03)	Very low	Very low certainty due to risk of bias (randomization concerns) and very serious imprecision (extremely wide confidence interval crossing multiple clinically important thresholds and small sample size).

Low-load resistance exercise vs Usual care	1	40	5.40 (-2.93, 13.73)	Very low	Very low certainty due to risk of bias (randomization concerns) and very serious imprecision (wide confidence interval crossing multiple clinically important thresholds and small sample size).
High-load resistance exercise vs Low-load resistance exercise	1	43	-6.80 (-15.83, 2.23)	Very low	Very low certainty due to risk of bias (randomization concerns) and very serious imprecision (extremely wide confidence interval crossing multiple clinically important thresholds and small sample size).
Exercise in water vs No intervention	1	25	-6.00 (-35.81, 23.81)	Very low	Very low certainty due to very serious risk of bias (high risk from protocol deviations and concerns in randomization) and very serious imprecision (extremely wide confidence interval crossing multiple clinically important thresholds and very small sample size).
Water exercise vs Pilates	1	68	8.73 (3.55, 13.91)	Low	Low certainty due to risk of bias (some concerns in randomization, protocol deviations, and outcome measurement) and serious imprecision (moderate sample size and wide confidence interval).
Clinical Pilates exercise vs Standard lymphedema exercises	1	60	1.67 (-0.81, 4.15)	Very low	Very low certainty due to risk of bias (randomization concerns) and very serious imprecision (confidence interval crossing multiple clinically important thresholds and limited sample size).
**Outcome: ROM – Shoulder extension < 6 months**					
High-load resistance exercise vs Usual care	1	41	-6.00 (-12.92, 0.92)	Very low	Very low certainty due to risk of bias (randomization concerns) and very serious imprecision (extremely wide confidence interval crossing multiple clinically important thresholds and small sample size).
Low-load resistance exercise vs Usual care	1	40	2.60 (-4.72, 9.92)	Very low	Very low certainty due to risk of bias (randomization concerns) and very serious imprecision (wide confidence interval crossing multiple clinically important thresholds and small sample size).
High-load resistance exercise vs Low-load resistance exercise	1	43	-8.60 (-15.30, -1.90)	Low	Low certainty due to risk of bias (randomization concerns) and serious imprecision (wide confidence interval covering different magnitudes of clinical effect and small sample size).
Water exercise vs Pilates	1	68	0.67 (-1.71, 3.05)	Very low	Very low certainty due to risk of bias (some concerns in randomization, protocol deviations, and outcome measurement) and very serious imprecision (confidence interval crossing multiple clinically important thresholds and limited sample size).
**Outcome: ROM – Shoulder abduction < 6 months**					
High-load resistance exercise vs Usual care	1	41	-8.30 (-20.72, 4.12)	Very low	Very low certainty due to risk of bias (randomization concerns) and very serious imprecision (extremely wide confidence interval crossing multiple clinically important thresholds and small sample size).
Low-load resistance exercise vs Usual care	1	40	0.60 (-8.28, 9.48)	Very low	Very low certainty due to risk of bias (randomization concerns) and very serious imprecision (extremely wide confidence interval crossing multiple clinically important thresholds and small sample size).
High-load resistance exercise vs Low-load resistance exercise	1	43	-8.90 (-19.74, 1.94)	Very low	Very low certainty due to risk of bias (randomization concerns) and very serious imprecision (extremely wide confidence interval crossing multiple clinically important thresholds and small sample size).
Clinical Pilates exercise vs Standard lymphedema exercises	1	60	3.67 (-2.82, 10.16)	Very low	Very low certainty due to risk of bias (randomization concerns) and very serious imprecision (wide confidence interval crossing multiple clinically important thresholds and moderate sample size).
Exercise in water vs No intervention	1	25	-14.40 (-45.49, 16.69)	Very low	Very low certainty due to very serious risk of bias (high risk from protocol deviations and concerns in randomization) and very serious imprecision (extremely wide confidence interval crossing multiple clinically important thresholds and very small sample size).
Water exercise vs Pilates	1	68	6.87 (2.50, 11.24)	Low	Low certainty due to risk of bias (some concerns in randomization, protocol deviations, and outcome measurement) and serious imprecision (confidence interval ranging from small to large clinical benefit and limited sample size).
**Outcome: ROM – Internal shoulder rotation < 6 months**					
Water exercise vs Pilates	1	68	1.93 (-1.05, 4.91)	Very low	Very low certainty due to risk of bias (some concerns in randomization, protocol deviations, and outcome measurement) and very serious imprecision (confidence interval crossing multiple clinically important thresholds and limited sample size).
**Outcome: ROM – External shoulder rotation < 6 months**					
Clinical Pilates exercise vs Standard lymphedema exercises	1	60	3.00 (-1.03, 7.03)	Very low	Very low certainty due to risk of bias (randomization concerns) and very serious imprecision (confidence interval crossing multiple clinically important thresholds and limited sample size).
Exercise in water vs No intervention	1	25	9.40 (-4.26, 23.06)	Very low	Very low certainty due to very serious risk of bias (high risk from protocol deviations and concerns in randomization) and very

					serious imprecision (extremely wide confidence interval crossing multiple clinically important thresholds and very small sample size).
Water exercise vs Pilates	1	68	0.67<br>(-3.16, 4.50)	Very low	Very low certainty due to risk of bias (some concerns in randomization, protocol deviations, and outcome measurement) and very serious imprecision (confidence interval crossing multiple clinically important thresholds and limited sample size).
**OUTCOME: Upper limb function (< 6 months)**					
Resistance exercise + CDT vs CDT	1	44	-6.40<br>(-16.22, 3.42)	Very low	Very low certainty due to risk of bias (randomization concerns) and very serious imprecision (extremely wide confidence interval crossing multiple clinically important thresholds and small sample size).
ALT vs home exercises	1	18	2.10<br>(-15.70, 19.90)	Very low	Very low certainty due to very serious risk of bias (high risk in randomization, protocol deviations, and outcome measurement) and very serious imprecision (extremely wide confidence interval crossing multiple clinically important thresholds and very small sample size).
Clinical Pilates exercise vs Standard lymphedema exercises	1	60	5.84<br>(-1.06, 12.74)	Very low	Very low certainty due to risk of bias (randomization concerns) and very serious imprecision (extremely wide confidence interval crossing multiple clinically important thresholds and small sample size).
